# Acarbose With Comparable Glucose-Lowering but Superior Weight-Loss Efficacy to Dipeptidyl Peptidase-4 Inhibitors: A Systematic Review and Network Meta-Analysis of Randomized Controlled Trials

**DOI:** 10.3389/fendo.2020.00288

**Published:** 2020-06-05

**Authors:** Fang Zhang, Shishi Xu, Lizhi Tang, Xiaohui Pan, Nanwei Tong

**Affiliations:** Division of Endocrinology and Metabolism, West China Hospital of Sichuan University, Chengdu, China

**Keywords:** acarbose, dipeptidyl peptidase-4 inhibitors, glucose-lowering efficacy, network meta-analysis, randomized controlled trials, tolerability and safety issue, type 2 diabetes, weight-loss effect

## Abstract

**Background:** Acarbose and dipeptidyl peptidase-4 inhibitors (DPP-4is) have several similarities regarding their efficacy. Assessing the hypoglycemic and weight-loss effects, as well as the tolerability between them at their optimal dosages, could provide a better management of adult type 2 diabetics.

**Methods:** We performed a systematic review and network meta-analysis (NMA) on randomized controlled trials that were identified from the databases of EMBASE, MEDLINE, Cochrane Central Register of Controlled Trials, Web of Science, Conference Proceedings Citation Index, ClinicalTrials.gov, China National Knowledge Infrastructure, Wan Fang, and SinoMed. The trials with 300 mg/day of acarbose or the recommended doses of DPP-4is were the most optimal for our NMA. The mean differences (MD) and relative risk (RR) derived from eligible studies were used.

**Results:** Among the 15,411 obtained potential studies, 13 pair-wise trials and 48 monotherapy studies were included in the meta-analysis and NMA, respectively. DPP-4is had a greater glucose-lowering effect, but a weaker weight-loss effect than acarbose in pair-wise meta-analysis (*p* < 0.05). However, NMA with 11,877 participants showed that, at their optimal doses, acarbose and DPP-4is had similar glucose-lowering effects on the 2-h postprandial glucose (MD 0.96 mmol/L, 95% credible interval −0.56 to 2.54), HbA1c (0.05%, −0.25 to 0.33), fasting plasma glucose reductions (−0.27 mmol/L, −0.76 to 0.24), and HbA1c < 7.0% target goal achievement (RR 1.33, 0.51 to 3.64). Acarbose was superior to DPP-4is regarding weight loss (MD −1.23 kg, −2.08 to −0.33). Acarbose had more withdrawal, gastrointestinal, and overall adverse events than DPP-4is (*p* < 0.05), but the differences disappeared after longer treatment (*p* > 0.05).

**Conclusions:** Acarbose and DPP-4is have similar glucose-lowering effects, but the weight-loss effects of acarbose are superior. Therefore, in the use of the most optimal dosages, overweight/obese type 2 diabetics might benefit more from a treatment with acarbose than DPP-4is.

## Introduction

Diabetes and its complications are approaching a global epidemic. However, the increasing glucose-lowering agents and international practice guidelines have dramatically improved the prognosis. Acarbose, the first approved α-glucosidase inhibitor (AGI), plays an essential role in delaying the glucose absorption from carbohydrate food. On the other hand, dipeptidyl peptidase-4 inhibitors (DPP-4is), which increase the levels of glucagon-like peptide-1 and gastric inhibitory polypeptide, stimulate the insulin secretion and reduce the blood sugar. Several recent guidelines for type 2 diabetes (T2D) management state that both AGIs and DPP-4is have moderate glucose-lowering efficacy, neutral weight impact and low risk of hypoglycemia (1, 2).

Acarbose and DPP-4is are two popular hypoglycemic agents, at least in East Asia. A growing evidence has focused on comparing between AGIs and DPP-4is (3–9). However, the pair-wise studies are quite limited, and the previous meta-analyses did not concentrate on acarbose. Additionally, a variety of new DPP-4is joined the market, which were not included in the previous studies. Therefore, the similarities and divergences between acarbose and DPP-4is remain ambiguous, and the uncertainty regarding their efficacy and tolerability sometimes makes it tricky for clinicians to choose the appropriate treatment option. To this end, we synthesized the available data from randomized controlled trials (RCT) to compare the glucose-lowering and weight-loss effects, as well as the safety issue between acarbose and DPP-4is at their recommended dosages. By combining the direct (comparing acarbose and DPP-4is within the same trials) and indirect (comparing them across trials with the same comparator) evidence, we conducted a systematic review and network meta-analysis (NMA) to further understand their benefit-risk profiles.

## Materials and Methods

### Search Strategy and Selection Criteria

We searched the databases of EMBASE, MEDLINE, Web of Science, Cochrane Central Register of Controlled Trials (CENTRAL), Conference Proceedings Citation Index, ClinicalTrials.gov (http://www.clinicaltrials.gov), SinoMed, Wan Fang, and China National Knowledge Infrastructure (CNKI) for relevant studies according to the following search query: T2D AND (acarbose AND/OR DPP-4is) AND RCT (specific search term shown in Supplementary Appendix). The last search was performed on September 1, 2018. There were no language restrictions for the literature search, and any additional study in the reference lists of identified trials or reviews was searched.

The inclusion criteria for the initially screened articles were: (i) the studies were RCTs with adult patients diagnosed with T2D; (ii) the intervention duration was at least 24 weeks; (iii) the sample size of each study was no less than 50 patients; (iv) the information on the key measures could be derived; and (v) the studies were published in English or Chinese. Besides, in order to maintain the consistency and transitivity of our NMA, further inclusion criteria should be addressed. First, the control intervention, which bridges the comparison between acarbose and DPP-4is, should be the same and referring to the specific regimens and their doses. Second, when the studies include combination therapies, the combined agents should also be exactly the same. Since we aimed to assess the benefits and potential risks at adequate-dosage therapy, the trials with 100 mg thrice a day of acarbose and/or the recommended doses of DPP-4is were the most optimal for our NMA. In addition, when a trial reported several phase results, we preferred the one with the longest duration and most qualified data. The duplication from the same trial was excluded. Three authors (FZ, LT, and XP) searched the literature, independently screened the title and abstract, and assessed the full text eligibility for identified trials. Any divergence in the opinions was resolved by discussion.

### Outcomes and Data Extraction

The primary aim of this study was to assess the differences in the glucose-lowering effect between acarbose and DPP-4is, especially the changes in the 2-h postprandial glucose (2hPG) and HbA1c. The secondary outcomes included the changes in the fasting plasma glucose (FPG) and body weight and numbers of participants who achieved the target goal of HbA1c < 7.0%, as well as the safety profiles. This study was conducted in accordance with the Preferred Reporting Items for Systematic Reviews and Meta-Analyses (PRISMA) statement and the PRISMA NMA extension statement (10, 11).

Two authors (FZ, SX) independently extracted the data, evaluated the study quality, and assessed the bias risk in the eligible articles. Any discrepancy was resolved by mutual checks or consensus of all the authors. The information of the first author name; publication year; study design; participants' racial heritages; sample size; interventions; treatment duration; mean diabetes duration; mean age; sex distribution; mean HbA1c and body mass index at the baseline; changes in the HbA1c, 2hPG, FPG, and body weight; number of participants who achieved HbA1c < 7.0%; and the number of patients who experienced adverse events (AE) were collected. Sufficient data were extracted from the original studies or calculated by the recommended methods (12).

### Assessments of Bias Risk and Quality

The quality of the included studies in the systematic review was evaluated by the Cochrane Collaboration tool, which is based on seven aspects (12). The three ranks in each category were low, high, and unclear risks.

### Data Synthesis and Statistical Analysis

The differences in the glucose-lowering efficacies and safety outcomes among the various interventions were detected. The data that were used in our meta-analysis were intended for treatment. A traditional pair-wise meta-analysis was performed to directly compare acarbose and DPP-4is in head-to-head studies using the Stata software version 14.0 (Stata Corp. College Station, TX, USA). The continuous outcomes were expressed as a standardized mean difference, and dichotomous data were represented as a relative risk (RR), both with a 95% confidence interval. The chi-square test and *I*^2^ statistics were used to evaluate the intertrial heterogeneity. If heterogeneity was detected (*p* ≤ 0.1; *I*^2^ > 50%), a random-effects model was applied; a fixed-effects model was adopted otherwise. When a statistical heterogeneity occurred, we initially analyzed its source, then performed a sensitivity analysis to examine the robustness. Regarding the 2hPG change outcome, if the heterogeneous results were robust, a meta-regression analysis was carried out to investigate the potential source. The publication bias was assessed using the funnel plot and Egger's linear regression test (13). In case a bias was present, the trim-and-fill computation was applied (14). A *p*-value < 0.05 was considered to be statistically significant unless otherwise stated.

In addition to the pair-wise meta-analysis, an NMA exploiting the direct and indirect comparisons was performed. We implemented a random-effect consistency model within a Bayesian framework in our NMA, using the “GeMTC” package version 0.14.3 of R software (version 3.4.0; R Foundation, Vienna, Austria) (15). The mean differences (MDs) or RRs with 95% credible intervals (CrI) were calculated using the Markov chain Monte Carlo methods, with Gibbs sampling based on 50,000 iterations after a burn-in phase of 20,000 iterations, when four Markov chains run simultaneously. The model convergence was evaluated according to the Brooks-Gelman-Rubin plots method (16), and the rank probabilities could be computed to obtain the hierarchy of each included treatment, which would contribute to the clinical use when significant variations are observed. In order to assess the NMA consistency, the node-splitting method was used, by means of reporting its Bayesian *p*-value to estimate whether the results between the direct and indirect evidence were consistent (17). Subgroup analyses were carried out according to the factors of the diabetes duration, treatment duration, different ethnicities of the participants, and individual DPP-4is.

## Results

### Literature Selection, Study Characteristics, and Quality of Bias Control

**Figure 1:**
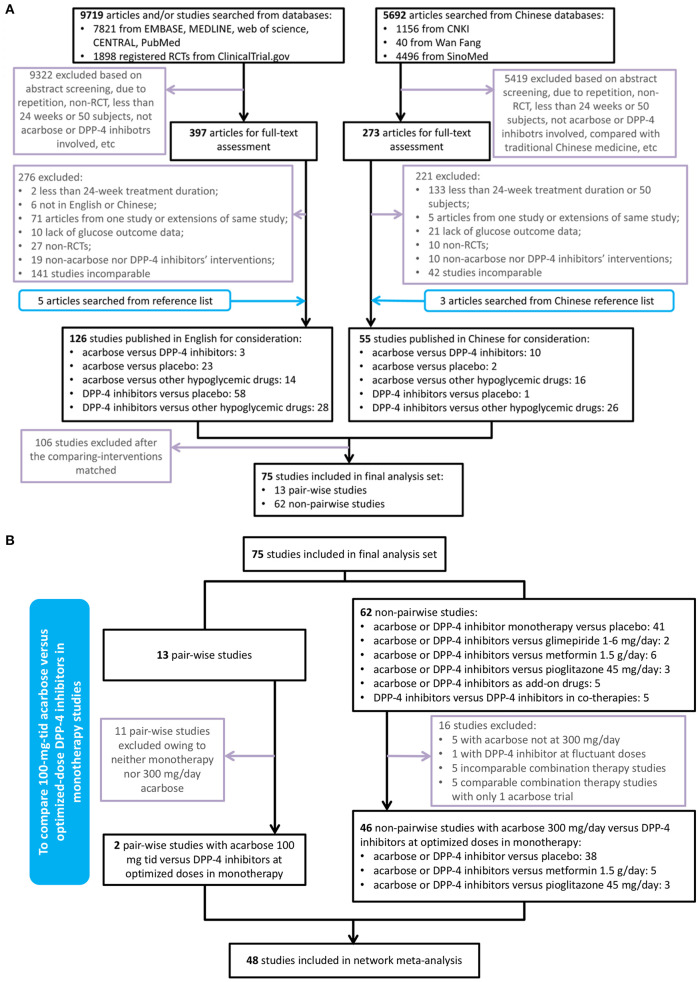
Flowchart of trial selection in the systematic review and network meta-analysis. **(A)** Flowchart of the 75 studies included in systematic review. **(B)** Flowchart of the 48 studies included in network meta-analysis. RCT, randomized controlled trial; DPP-4, dipeptidyl peptidase-4; TID, thrice a day.

A total of 15,411 studies were identified as a result of the electronic search, among which 9,719 articles were published in English and 5,692 in Chinese. After initial screening, 14,741 studies were excluded, and 670 trials were retrieved for detailed assessment. After full-text screening, 173 publications and 8 additional studies through manual search were potentially eligible for our systematic review. Among them, 13 pair-wise studies directly comparing acarbose and DPP-4is were included in our meta-analysis (Table S1). After matching all the comparators in non-head-to-head trials, 106 publications were excluded due to their incomparability (Figure 1A). Five comparable studies with metformin-combined therapy were also excluded since only one acarbose trial was included. Therefore, all the studies included in our NMA were monotherapy, which could maintain and improve the transitivity. Since our aim was to compare acarbose and DPP-4is at their optimized dosages, 48 monotherapy studies were finally eligible for our NMA (Figure 1B). Therefore, 75 RCTs with 21,806 participants were included in our analysis set (Table S2), while 59 studies with 13,322 patients were obtained for the meta-analysis and NMA.

The characteristics of the 59 studies are described in Table S1. Our meta-analysis and NMA involved 994 and 2,388 individuals with acarbose, along with 1,211 and 4,314 participants with DPP-4is, respectively. The quality assessment of the 75 RCTs regarding the bias risks is displayed in Table S3, which shows that most of them showed moderate-to-high qualities.

### Pair-Wise Meta-Analysis

We divided the 13 head-to-head studies into three subgroups according to the treatments: monotherapy (*n* = 3), co-therapy with oral anti-diabetic drugs (OAD) (*n* = 6), and co-therapy with insulin (*n* = 4). The results of the funnel plots and Egger's tests revealed that no potential publication bias existed across the included studies (Figure S1).

**Figure 2:**
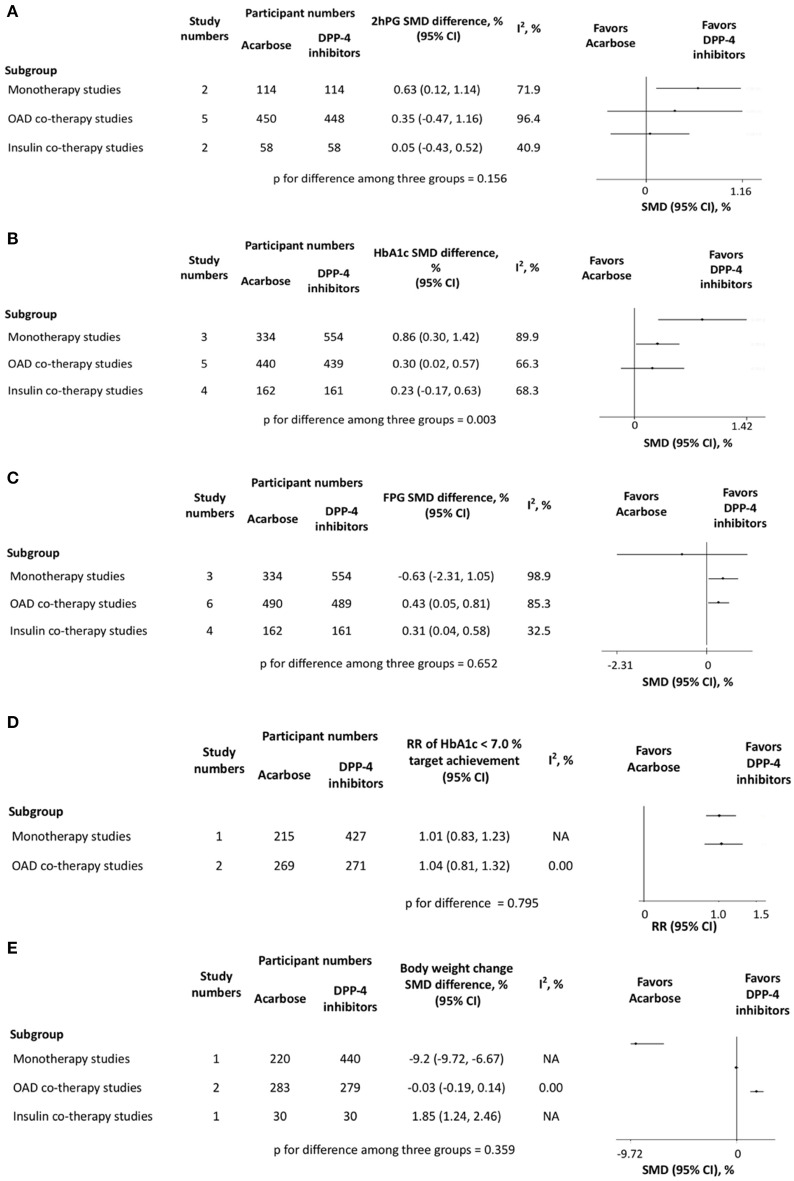
Pair-wise meta-analysis for comparisons between acarbose and dipeptidyl peptidase-4 inhibitors in **(A)** 2-h postprandial glucose change, **(B)** HbA1c change, **(C)** fasting plasma glucose change, **(D)** relative risk of HbA1c < 7.0% target achievement and **(E)** body weight change. DPP-4, dipeptidyl peptidase-4; OAD, oral anti-diabetic drug; SMD, standardized mean difference; CI, confidence interval; 2hPG, 2-h postprandial glucose; FPG, fasting plasma glucose; RR, relative risk.

In most of the comparisons, DPP-4is seemed to have a better glucose-lowering effect than acarbose. The 2hPG reduction in DPP-4is was superior to that in acarbose in the monotherapy group (*p* < 0.05) (Figure 2A). Both the reductions in HbA1c and FPG were larger in DPP-4is than those in acarbose (Figures 2B,C). However, the acarbose monotherapy had a greater weight-loss effect than the vildagliptin monotherapy (*p* < 0.05) (Figure 2E). Intertrial heterogeneity was detected in the 2hPG change. Both sensitivity analysis (Figure S2) and trim-and-fill procedure (*p* = 0.504) showed no obvious low-quality trial. The univariate meta-regression analysis suggested that various acarbose dosages (*p* = 0.011) and treatment durations (*p* = 0.000) were significantly correlated with the results (Table S4).

The RRs of the AEs are depicted in Figure S3. Compared with DPP-4is, acarbose led to more gastrointestinal (Figure S3C) and overall AEs (Figure S3E) in the monotherapy group (*p* < 0.05). However, there were no significant differences in the RRs of withdrawal (Figure S3A), serious AEs (Figure S3B), or hypoglycemia events (Figure S3D).

Due to the various acarbose dosages weakening the consistency of the studies in the pair-wise meta-analysis, we conducted an NMA to further compare acarbose with DPP-4is at their recommended dosages.

### Bayesian Network Meta-Analysis

#### Comparisons of the Glucose-Lowering and Weight-Loss Effects

**Figure 3:**
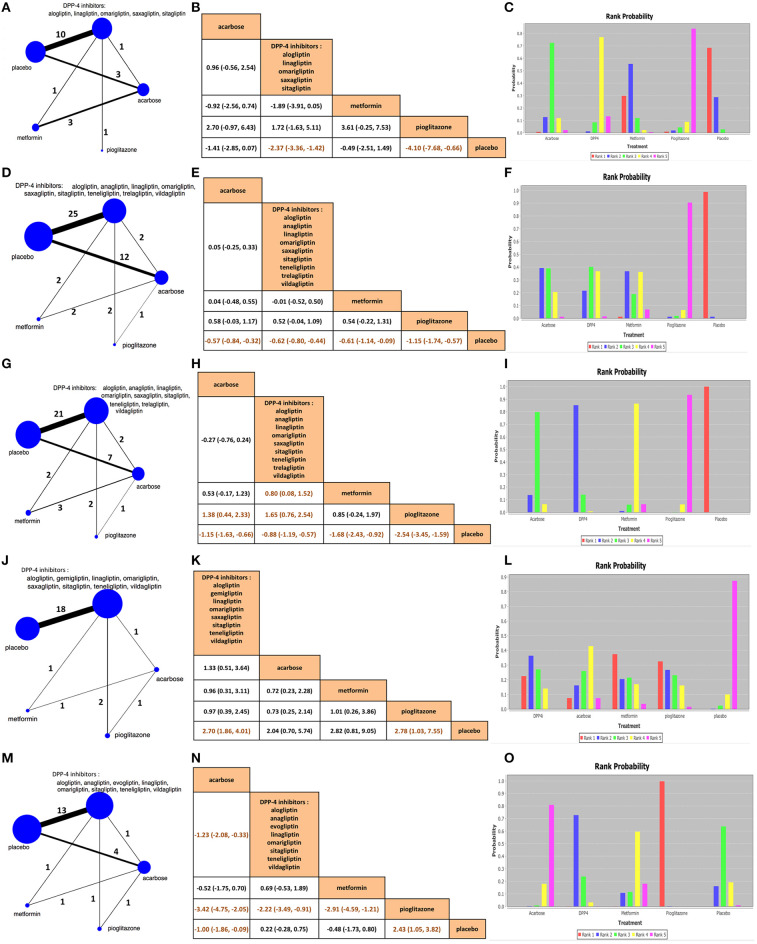
Network plots, network meta-analysis comparisons, and rank probabilities among acarbose, dipeptidyl peptidase-4 inhibitors, metformin, pioglitazone, and placebo in **(A–C)** 2-h postprandial glucose change, **(D–F)** HbA1c change, **(G–I)** fasting plasma glucose change, **(J–L)** relative risk of HbA1c < 7.0% target achievement, and **(M–O)** body weight change. In the network plots, the size of the nodes corresponds to the number of participants assigned to each treatment. Numbers by the lines indicate the cumulative number of enrolled studies for each direct comparison. The dark orange letters in the comparison tables imply significant differences. DPP-4, dipeptidyl peptidase-4.

Our NMA involved 48 monotherapy trials, whose comparators included acarbose (300 mg/day), DPP-4is, metformin (1,500 mg/day), pioglitazone (45 mg/day), and placebo. The 2hPG reduction was reported in 18 studies with 3,534 participants, showing that DPP-4is and pioglitazone were better than placebo (*p* < 0.05), but no significant difference was observed between acarbose and DPP-4is (MD 0.96 mmol/L, 95% CrI −0.56 to 2.54) (Figures 3A–C). On the other hand, 45 trials with 8,974 patients verified that all the active hypoglycemic drugs were better than placebo at HbA1c reduction (*p* < 0.05) (Figures 3D–F). Considering the FPG decrease, 37 studies with 7,683 individuals showed that pioglitazone had the best effect (MD −2.54 mmol/L, 95% CrI −3.45 to −1.59), followed by metformin (−1.68, −2.43 to −0.92), acarbose (−1.15, −1.63 to −0.66), DPP-4is (−0.88, −1.19 to −0.57), and placebo in the rank order (Figures 3G-I). The RRs of HbA1c < 7.0% target achievement were reported by 23 trials with 6,620 participants, implying that DPP-4is (2.70, 95% CrI 1.86–4.01) and pioglitazone (2.78, 1.03–7.55) were superior to placebo (Figures 3J–L). Notably, based on the 21 trials with 4,062 patients, acarbose (MD −3.42 kg, 95% CrI −4.75 to −2.05) had the most efficacy for weight loss compared with pioglitazone, followed by metformin (−2.91, −4.59 to −1.21), placebo (−2.43, −3.82 to −1.05), and DPP-4is (−2.22, −3.49 to −0.91) (Figures 3M–O).

There were seven trials with a treatment duration of more than 48 weeks. The HbA1c (*n* = 715, Figures S4A,B) and FPG (*n* = 509, Figures S4C,D) changes were not significantly different among acarbose, DPP-4is, and placebo. However, the weight loss effect (*n* = 246) was more obvious in DPP-4is than that in placebo (MD −0.80 kg, 95% CrI −1.51 to −0.12) (Figures S4E,F).

The patients in 20 studies were mostly Asians (with a percentages of 90.5–100.0%). Acarbose displayed equal effects to those of DPP-4is in the 2hPG (*n* = 2,357, Figures S4G,H), HbA1c (*n* = 4,936, Figures S4I,J), weight reductions (*n* = 2,475, Figures S4O,P), as well as the HbA1c < 7.0% achievement (*n* = 3,609, Figures S4M,N). It is worth mentioning that acarbose was superior to DPP-4is at FPG reduction in Asian patients (*n* = 3,903, MD −0.81 mmol/L, 95% CrI −1.50 to −0.09) (Figures S4K,L).

The mean diabetes durations of the participants in five studies were more than 5 years. Due to the lack of acarbose relevant studies, the comparison was only observed in the HbA1c change (*n* = 881), where acarbose probably had a weaker effect than that of DPP-4is (Figures S4Q,R).

Our study included 38 placebo-controlled trials, whose comparators included the medicines of acarbose, alogliptin, anagliptin, evogliptin, gemigliptin, linagliptin, omarigliptin, saxagliptin, sitagliptin, teneligliptin, trelagliptin, and vildagliptin. Regarding the 2hPG reduction, sitagliptin (MD −2.88 mmol/L, 95% CrI −4.40 to −1.30) was better than placebo, while sitagliptin and omarigliptin were probably more effective than acarbose (*n* = 2,775) (Figures S5A–C). Acarbose (MD −0.55%, 95% CrI −0.83 to −0.28), linagliptin (−0.64, −1.11 to −0.15), omarigliptin (−0.85, −1.68 to −0.01), sitagliptin (−0.91, −1.27 to −0.57), and vildaglipitn (−0.53, −0.98 to −0.11) had significant improvements in the HbA1c reduction compared with placebo, such that sitagliptin, omarigliptin, and linagliptin seemed more efficient than acarbose (*n* = 7,926) (Figures S5D–F). On the other hand, acarbose (MD −1.14 mmol/L, 95% CrI −1.78 to −0.48), linagliptin (−1.04, −2.08 to 0.00), and sitagliptin (−0.97, −1.68 to −0.27) had greater effects on the FPG reduction than placebo, such that acarbose possibly had the greatest effect (*n* = 6,046) (Figures S5G–I). The RRs of the HbA1c < 7.0% achievement showed that, compared with placebo, acarbose (4.23, 95% CrI 1.05–17.86), alogliptin (2.10, 1.06–4.39), gemigliptin (3.37, 1.16–9.83), omarigliptin (11.78, 3.70–42.19), sitagliptin (2.83, 1.73–4.73), teneligliptin (9.11, 2.82–31.46), and vildagliptin (4.15, 1.40–13.00) were more effective. Omarigliptin and teneligliptin probably had the highest rank, followed by acarbose, then vildagliptin, gemigliptin, sitagliptin, and alogliptin in the rank order (*n* = 4,789) (Figures S5J–L). Although it was surprising to find no significant difference in the weight-loss effect among acarbose, DPP-4is and placebo, acarbose tended to have the highest rank among them (*n* = 2,884) (Figures S5M–O).

The baseline characteristics of all the 48 studies that were included in the NMA were assessed, and only three of them reported the baseline HbA1c or/and body weight with significant differences within the trials. The baseline comparisons for the hypoglycemic and weight-loss results of NMA are shown in Figure S6. Based on the baseline situation, the above-mentioned NMA findings are robust.

#### Safety Comparisons

**Figure 4:**
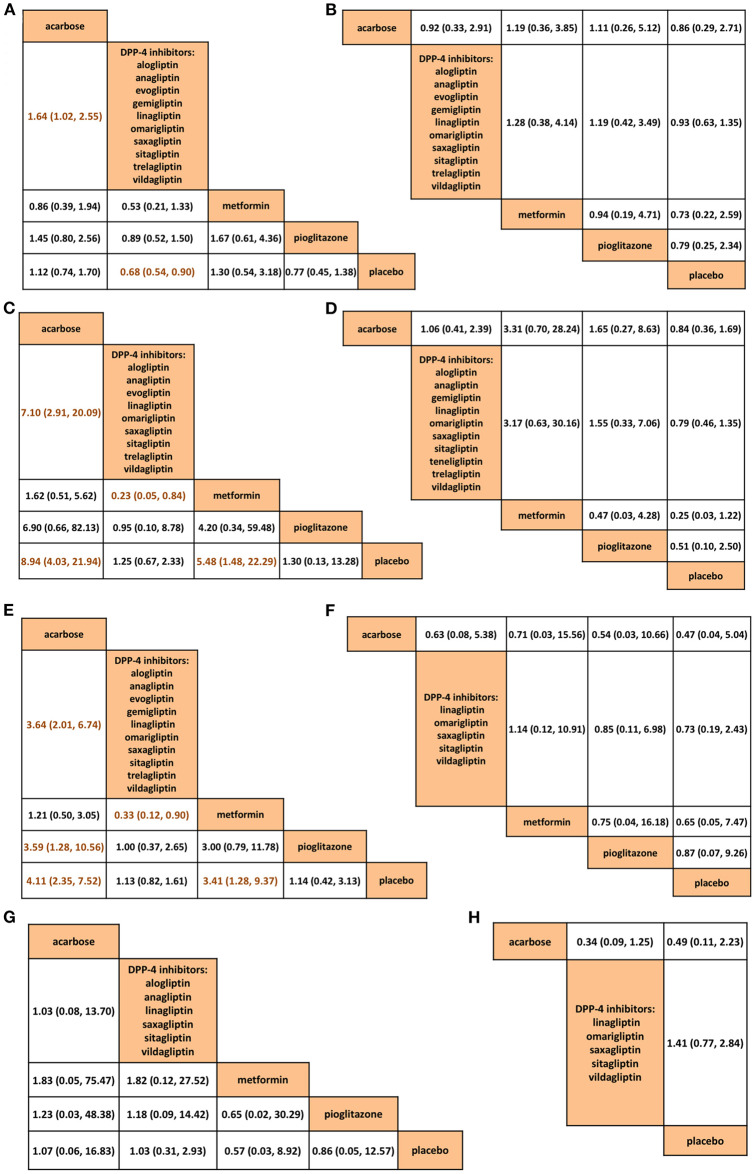
Comparisons among acarbose, dipeptidyl peptidase-4 inhibitors, metformin, pioglitazone, and placebo in network meta-analysis in **(A)** withdrawal, **(B)** serious adverse events, **(C)** gastrointestinal side effects, **(D)** hypoglycemia, **(E)** overall adverse events, **(F)** nasopharyngitis, **(G)** dizziness, and **(H)** upper respiratory tract infection. The dark orange letters in the comparison tables suggest significant differences. DPP-4, dipeptidyl peptidase-4.

The safety issues, including withdrawal (*n* = 9,881) (Figure 4A), serious AE (*n* = 8,779) (Figure 4B), gastrointestinal side effects (*n* = 6,566) (Figure 4C), hypoglycemia (*n* = 9,179) (Figure 4D), overall AEs (*n* = 8,982) (Figure 4E), nasopharyngitis (*n* = 2,199) (Figure 4F), dizziness (*n* = 3,194) (Figure 4G), and upper respiratory infection (*n* = 2,175) (Figure 4H) were compared across all the interventions (rank probabilities are shown in Figure S7). DPP-4is were superior to acarbose in withdrawal (RR 0.61, 95% CrI 0.39–0.98), gastrointestinal (0.14, 0.05–0.34) and overall AEs (0.27, 0.15–0.50). Interestingly, the disparities between acarbose and DPP-4is disappeared after longer treatment (*n* = 277–679) (Figure S8). (The AE data collected across all the 48 studies are shown in Table S5)

#### Comparisons Between Direct and Indirect Evidence

The node-splitting analysis was used to assess the consistency between the direct and indirect evidence. Except for the results of the Asian subgroup analyses in the 2hPG (Figure S9A) and FPG (Figure S9B) changes, all the other outcomes were consistent. Although the inconsistency limited the use of these results, the consistent findings of the other calculations verified the reliability and transitivity of our NMA.

## Discussion

To the best of our knowledge, this is the largest NMA comparing acarbose with DPP-4is, overcoming the limited source of head-to-head trials. According to our pair-wise meta-analysis, DPP-4is seemed superior to acarbose with regard to the glucose-lowering effect, whereas acarbose provided a greater weight reduction effect than vildagliptin. In order to abate the interferences that result from diverse acarbose dosages and multiple therapy designs, we narrowed down our included trials in the NMA to monotherapy, such that only the trials with acarbose of 300 mg/day or DPP-4is at their recommended dosages remained. Accordingly, the glucose-lowering efficacies between the two drugs were almost comparable, while compared with DPP-4is, the patients benefited more from acarbose regarding the weight loss, but had more risks considering the withdrawal and gastrointestinal and overall side effects. Intriguingly, with the use of their most optimal dosages, the differences between the two drugs disappeared after longer treatment.

Acarbose slows the carbohydrate absorption, and its hypoglycemic effect was only considered for decreasing the postprandial glucose excursions (18, 19). However, acarbose was recently observed to decrease the fasting glucose and contribute to maintaining stable glucose levels (4, 20, 21). On the other hand, DPP-4is are believed to reduce the postprandial glucose fluctuations by improving the incretins response to meal ingestion, and they also reduce the fasting glucose via inhibiting the hepatic glucose output (22). Thereby, both acarbose and DPP-4is belong to the category of OADs, not only reducing the temporary glucose level but also stabilizing the glycemic variability.

Our NMA revealed that compared with placebo, acarbose and DPP-4is had significant hypoglycemic effects, since they both have a comparable glucose-lowering efficacy with their optimal doses. This result is quite different from our own and previous meta-analyses, suggesting that DPP-4is are superior to AGIs at glucose reduction (3, 5, 7). The reasons for the inconsistency may be as follows: Firstly, due to the limited source of pair-wise trials, one study, respectively, compared AGIs or DPP-4is with placebo, so the results may mislead the readers (7). Secondly, the previous meta-analyses included only few acarbose studies, which were not sufficient to draw a representative conclusion (3, 5). Thirdly, the dosages of acarbose varied quite differently in the existed trials, while a dose of 300 mg/day has been shown to be the most efficacious worldwide (23). Thus, using the most recommended dose to evaluate the efficacy and tolerability of acarbose is a reasonable way for its assessment.

In order to obtain the best results, we chose a sufficiently long intervention duration in this study, whose included trials were of at least 24-week periods. Surprisingly, we observed no significant hypoglycemic differences among acarbose, DPP-4is and placebo after 48-week treatment. Acarbose could consistently contribute to the glucose reduction for 3–5 years (24, 25), while DPP-4is have a durability of 3–4 years (26–29). Although both acarbose and DPP-4is in our NMA showed a relatively short sustained glucose-lowering efficacy, they were probably still better than placebo.

The main mechanism of acarbose to optimize the glucose metabolism is the role in slowing the postprandial glucose absorption, which is almost independent from the insulin secretion or its action (30). Accordingly, acarbose could be a convenient option for the long-term treatment irrespective of the diabetes duration. Conversely, DPP-4is ultimately improve the glucose regulation by affecting the insulin secretion as insulin secretagogues, whose hypoglycemic efficacy may be attenuated by the disease progression (29, 31, 32). Our NMA suggested that DPP-4is are probably better than acarbose at HbA1c reduction in the patients with long-term diabetes, but only one acarbose trial was included. Further studies are thus needed to determine whether the hypoglycemic effects are maintained over the years, especially among the patients with a long disease progression.

Acarbose and DPP-4is were reported to have a higher efficacy in Eastern patients than in the Western counterparts (23, 33, 34). The underlying mechanisms are linked to the different dietary habits, insulin responses, BMI levels and genetic diversities. Our NMA depicted that acarbose and DPP-4is had equal effects among the Asian populations, except for the fact that acarbose had a greater FPG reduction effect than DPP-4is. However, due to the inconsistency in the FPG results between the direct and indirect comparisons, these findings could not be extrapolated, and they need to be further studied.

A total of 11 different DPP-4is were included in our NMA. Due to their various molecular skeletons, pharmacological characteristics and dosing schedules, different DPP-4is could display widely divergent results when compared with acarbose. As expected, based on the placebo-controlled studies, some DPP-4is were better while the others were weaker than acarbose considering their efficacies. The medicines of acarbose, sitagliptin, linagliptin, alogliptin and vildagliptin were superior to placebo regarding the glucose reduction effect, while sitagliptin and linagliptin had no significant weight change effect. These findings of DPP-4is were in accordance with the previous studies (35, 36). With the emergence of various new DPP-4is, our NMA hinted at the diversities across the individual ones although the limited source of pair-wise trials could not allow for a direct comparison among them.

Plenty of evidence has demonstrated that acarbose and DPP-4is at least have a weight-neutral effect (1, 2, 19, 37). In consistence with the previous meta-analyses, acarbose was associated with a significant weight reduction compared with DPP-4is in our NMA (3, 5). In accordance with previous studies, alogliptin probably had a greater weight reduction effect than linagliptin, sitagliptin, and vildagliptin (35, 36). However, the positive weight impact in our NMA disappeared after longer intervention. A real-world study confirmed that the weight reduction effect of acarbose was sustainable over 5 years (24), whereas we need to identify the long-term weight effect of DPP-4is. Additionally, some factors, such as the initial glucose level, may influence the weight change effect of antidiabetic agents and needs further clarification (38). Since weight management is a pivotal aspect of T2D treatment, in the use of the recommended dosage, acarbose might be more favorable to overweight/obese diabetics in light of its weight loss effect.

The gastrointestinal side effects are common in acarbose, but some studies showed that the discomforts could be improved with a gradual titration to the maintenance dosage and fiber-rich nutritional dietary (23, 39). Although an increased evidence is associating DPP-4is with the effects of nasopharyngitis, dizziness or upper respiratory infection, there is no evidence of a DPP-4is-related weakened immune response (22, 40). Interestingly, the AE differences disappeared after a longer treatment in our NMA, implying that the safety profiles of acarbose and DPP-4is may be similar, especially when the dosages are tolerable.

Our study has several limitations. Firstly, we did not retrieve the cardiovascular outcomes (CVOs). Since the aim was to concentrate on the hypoglycemic efficacies of acarbose and DPP-4is, our included trials were narrowed down to monotherapy, while several CVO trials, such as the SAVOR-TIMI 53, TECOS, EXAMINE, and CARMELINA studies, had patients with long diabetes durations who were receiving combined hypoglycemic therapies (26–28, 41). Consequently, our included studies cannot form a base to draw a CVO conclusion. Secondly, due to the rapid emergence of involving evidence, it is difficult to capture all the relevant literatures, which led to choosing a specific time to end the selection. Thirdly, some inconsistencies existed in our NMA, restricting the generalizability of those results. As a result, further head-to-head studies are needed to assess the efficacy and tolerability as well as the cardiovascular safety between acarbose and DPP-4is in long-term clinical use.

## Conclusion

In summary, our findings suggested that when used at the optimal doses, acarbose has a comparable glucose-lowering efficacy to that of DPP-4is, but it is superior in the weight loss effect. Moreover, there is no significant difference in the safety issues after a sufficiently long intervention period. Therefore, compared with DPP-4is, acarbose might be more beneficial in overweight/obese T2D patients with their recommended dosages. Although further pair-wise trials are required to examine our findings in long-term practice, we hope that the presented analysis contributes a helpful prospective to the clinical use.

## Data Availability Statement

All datasets generated for this study are included in the article/Supplementary Material.

## Author Contributions

FZ and NT obtained funding for the study and designed it. FZ, LT, and XP participated in literature searches and study selection. FZ and SX extracted data, evaluated study quality, and assessed bias risk of eligible trials. FZ and XP carried out all statistical analyses. FZ and SX interpreted the data and FZ drafted the report. NT revised the manuscript critically. All the authors contributed to resolve divergence and approved the final submitted version. The corresponding author has full access to all data in the study and takes final responsibility for the decision to submit for publication.

## Conflict of Interest

The authors declare that the research was conducted in the absence of any commercial or financial relationships that could be construed as a potential conflict of interest.
