# Supplementary material for: Acarbose With Comparable Glucose-Lowering but Superior Weight-Loss Efficacy to Dipeptidyl Peptidase-4 Inhibitors: A Systematic Review and Network Meta-Analysis of Randomized Controlled Trials
Source: Front Endocrinol (Lausanne). 2020 Jun 5;11:288. doi: 10.3389/fendo.2020.00288 (PMC7291873; doi:10.3389/fendo.2020.00288)
Supplement: Supplementary file 1 [file Data_Sheet_1.PDF]

Supplementary Appendix and Figures

I. Specific search term:

(1) type 2 diabetes:

(non insulin dependent diabetes mellitus) OR [(typ? 2 or typ? II or typ?2 or typ?II.tw,ot.) AND (diabet$.tw,ot)]

(2) acarbose:

Acarbose OR Precise OR Glumida OR Glucobay OR Prandase OR Glibose OR Glicobase OR Gluconase OR Glucor OR Rebose OR (Glycoside Hydrolase Inhibitor?) OR (glucosidase inhibitor?)

(3) dipeptidyl peptidase-4 inhibitors:

(Dipeptidyl Peptidase IV Inhibitor) OR (Dipeptidyl Peptidase 4 Inhibitor) OR (DDP-4 inhibitor?) OR (DPP-IV Inhibitor) OR Sitagliptin OR Januvia OR Linagliptin OR Tradjenta OR Alogliptin OR Nesina OR Saxagliptin OR Onglyza OR Vildagliptin OR Galvus OR *gliptin

(4) randomized controlled trial：

(Randomized Controlled Trial) OR (Controlled Clinical Trial) OR (Clinical Trial) OR (Comparative Study) OR (Drug comparison) OR (Crossover procedure) OR (Double blind procedure) OR (Single blind procedure)

Search strategy: (1) AND [(2) AND/OR (3)] AND (4)

II. Reference list of the 75 trials included in our systematic review (in the order in **Table S2**)

1. Pan C, Yang W, Barona JP, Wang Y, Niggli M, Mohideen P, Wang Y, Foley JE. Comparison of vildagliptin and acarbose monotherapy in patients with type 2 diabetes: a 24-week, double-blind, randomized trial. Diabet Med 2008; **25**(4): 435-441.

2. Wang MM, Lin S, Chen YM, Shu J, Lu HY, Zhang YJ, Xie RY, Zeng LY, Mu PW. Saxagliptin is similar in glycaemic variability more effective in metabolic control than acarbose in aged type 2 diabetes inadequately controlled with metformin. Diabetes Res Clin Pract 2015; **108**(3): e67-70.

3. Du J, Liang L, Fang H, Xu F, Li W, Shen L, Wang X, Xu C, Bian F, Mu Y. Efficacy and safety of saxagliptin compared with acarbose in Chinese patients with type 2 diabetes mellitus uncontrolled on metformin monotherapy: Results of a Phase IV open-label randomized controlled study (the SMART study). Diabetes Obes Metab 2017; **19**(11): 1513-1520.

4. Bao Y, Chen G. Efficacy of acarbose and sitagliptin on type-2 diabetes and influence on gastrointestinal related hormones. Practical Pharmacy And Clinical Remedies 2014; **17**(8): 966-969.

5. Guo F, Mao H, Wu X, Liu X, Ma X, Wu L, Gao S. Observation on effect of sitagliptin phosphate for treating 52 cases of early diabetic nephropathy. China Pharmaceuticals 2015; **24**(9): 12-13.

6. Li G, Wang S, Qi T, Ma L, Niu Y. Analysis of the effect of sitagliptin on the clinical efficacy and renal function of patients with type 2 diabetes mellitus. Chiese Journal of the Frontiers of Medical Science (Electronic Version) 2016; **8**(9): 169-172.

7. Xue X. Influence of sitagliptin to carotid intima-media thickness of patients with type 2 diabetes. China Medicine And Pharmacy 2016; **6**(16): 64-67.

8. Zhang K, Ren Q, Wu T, Du J. Effect of sitagliptin on markers of risk factors and risk factors correlated with cardiovascular complications of type 2 diabetes. Clinical Misdiagnosis & Mistherapy 2016; **29**(5): 94-98.

9. Gao J, Li X, Wang M. The effectiveness and safety of saxagliptin combined with insulin glargine in the treatment of gerontal patients with type 2 diabetes mellitus. Chiese Journal of the Frontiers of Medical Science (Electronic Version) 2015; **7**(8): 29-31.

10. Su G. Effects of glargine combined with saxagliptin on blood glucose control of type 2 diabetic patient. Medical Innovation of China 2016; **13**(35): 39-42.

11. Li D. The effect observation of metformin combined with alogliptin in the treatment of type 2 diabetes. Medical Journal of Chinese People's Health 2016; **28**(5): 46-47.

12. Zhang Y. The effect observation of metformin and alogliptin combined with pioglitazone in the treatment of type 2 diabetes. Chinese and Foreign Medical Research 2016; **14**(5): 13-15.

13. Duan J, Peng D, Dong B, Liu F, Mao L. Effect of sitagliptin plus melbine on blood glucose and incidence rate of cardiovascular events in patients with type 2 diabetes mellitus. Journal of Clinical Medicine in Practice 2016; **20**(5): 18-21.

14. Hanefeld M, Fischer S, Schulze J, Spengler M, Wargenau M, Schollberg K, Fücker K. Therapeutic potentials of acarbose as first-line drug in NIDDM insufficiently treated with diet alone. Diabetes Care 1991; **14**(8): 732-737.

15. Coniff RF, Shapiro JA, Seaton TB, Bray GA. Multicenter, placebo-controlled trial comparing acarbose (BAY g 5421) with placebo, tolbutamide, and tolbutamide-plus-acarbose in non-insulin-dependent diabetes mellitus. Am J Med 1995; **98**(5): 443-451.

16. Hoffmann J, Spengler M. Efficacy of 24-week monotherapy with acarbose, glibenclamide, or placebo in NIDDM patients. The Essen Study. Diabetes Care 1994; **17**(6): 561-566.

17. Hoffmann J, Spengler M. Efficacy of 24-week monotherapy with acarbose, metformin, or placebo in dietary-treated NIDDM patients: the Essen-II Study. Diabetes Care 1997; **103**(6): 483-490.

18. Chan JC, Chan KW, Ho LL, Fuh MM, Horn LC, Sheaves R, Panelo AA, Kim DK, Embong M. An Asian multicenter clinical trial to assess the efficacy and tolerability of acarbose compared with placebo in type 2 diabetic patients previously treated with diet. Asian Acarbose Study Group. Diabetes Care 1998; **21**(7): 1058-1061.

19. Holman RR, Cull CA, Turner RC. A randomized double-blind trial of acarbose in type 2 diabetes shows improved glycemic control over 3 years (U.K. Prospective Diabetes Study 44). Diabetes Care 1999; **22**(6): 960-964.

20. Josse RG, Chiasson JL, Ryan EA, Lau DC, Ross SA, Yale JF, Leiter LA, Maheux P, Tessier D, Wolever TM, Gerstein H, Rodger NW, Dornan JM, Murphy LJ,

Rabasa-Lhoret R, Meneilly GS. Acarbose in the treatment of elderly patients with type 2 diabetes. Diabetes Res Clin Pract 2003; **59**(1): 37-42.

21. Kirkman MS, Shankar RR, Shankar S, Shen C, Brizendine E, Baron A, McGill J. Treating postprandial hyperglycemia does not appear to delay progression of early type 2 diabetes: the Early Diabetes Intervention Program. Diabetes Care 2006; **29**(9): 2095-2101.

22. Wolever TM, Chiasson JL, Josse RG, Hunt JA, Palmason C, Rodger NW, Ross SA, Ryan EA, Tan MH. Small weight loss on long-term acarbose therapy with no change in dietary pattern or nutrient intake of individuals with non-insulin-dependent diabetes. Int J Obes Relat Metab Disord 1997; **21**(9): 756-763.

23. 征革凡，王金平， 张辉， 胡泽溪， 刘娟， 肖建中， 陈仕明， 曹辉碧， 李光伟， 胡英华， 潘孝仁. 拜糖平治疗非胰岛素依赖型糖尿病的临床观察. 中华内分泌代谢杂志 1995; **11**(3): 163-164.

24. Fischer S, Hanefeld M, Spengler M, Boehme K, Temelkova-Kurktschiev T. European study on dose-response relationship of acarbose as a first-line drug in non-insulin-dependent diabetes mellitus: efficacy and safety of low and high doses. Acta Diabetol 1998; **35**(1): 34-40.

25. Wu G, Han Y, Yu Y, Teng X, Zhang J, Qu F. Influence of acarbose on insulin resistant of patients with type 2 diabetes mellitus. Chin J New Drugs Clin Rem 2003; **22**(9): 535-538.

26. Hasche H, Mertes G, Bruns C, Englert R, Genthner P, Heim D, Heyen P, Mahla G, Schmidt C, Schulze-Schleppinghof B, Steger-Johannsen G. Effects of acarbose treatment in Type 2 diabetic patients under dietary training: a multicentre, double-blind, placebo-controlled, 2-year study. Diabetes Nutr Metab 1999; **12**(4): 277-285.

27. Braun D, Schonherr U, Mitzkat H. Efficacy of acarbose mono therapy in patients with type 2 diabetes: a double-blind study conducted in general practice. Endocrinology and Metabolism 1996; **3**: 275-280.

28. Kovacevic I, Profozic V, Skrabalo Z, Cabrijan T, Zjacic-Rotkvic V, Goldoni V, Jovic-Paskvalin Lj, Crncevic-Orlic Z, Koselj M, Metelko Z. Multicentric clinical trial to assess efficacy and tolerability of acarbose (Bay g 5421) in comparison to glibenclamide and placebo. Diabetologia Croatica 1997; **26**(2): 83-89.

29. 胡寒静，雷琳，赵枝琴. 格列美脲和阿卡波糖治疗新诊断 2 型糖尿病疗效对比分析. 贵阳中医学院学报 2012; **5**(34): 70-71.

30. Yang W, Liu J, Shan Z, Tian H, Zhou Z, Ji Q, Weng J, Jia W, Lu J, Liu J, Xu Y, Yang Z, Chen W. Acarbose compared with metformin as initial therapy in patients with newly diagnosed type 2 diabetes: an open-label, non-inferiority randomised trial. Lancet Diabetes Endocrinol 2014; **2**(1): 46-55.

31. 王连英，李玉凤，赵翠伶，郭光霞. 二甲双胍缓释片和阿卡波糖治疗新诊断 2 型糖尿病疗效对比分析. 中国医刊 2011; **46**(7): 57-58.

32. 荣佐民. 二甲双胍和拜糖平对 2 型糖尿病降糖作用的临床疗效观察. 中国医药指南 2008; **6**(4): 18-19.

33. Zhu Z, Qiu X, Zhu H, Ding Z. Application of acarbose combined with metformin in treatment of newly diagnosed type 2 diabetes patients. Heilongjiang Medicine Journal 2011; **24**(2): 223-225.

34. Göke B, German Pioglitazone Study Group. Improved glycemic control and lipid profile in a randomized study of pioglitazone compared with acarbose in patients with type 2 diabetes mellitus. Treat Endocrinol 2002; **1**(5): 329-336.

35. A Y. The effects of acarbose combined with metformin on blood glucose and blood fat of patients with type 2 diabetes. Diabetes New World 2015; **11**: 25-27.

36. DeFronzo RA, Fleck PR, Wilson CA, Mekki Q; Alogliptin Study 010 Group. Efficacy and safety of the dipeptidyl peptidase-4 inhibitor alogliptin in patients with type 2 diabetes and inadequate glycemic control: a randomized, double-blind, placebo-controlled study. Diabetes Care 2008; **31**(12): 2315-2317.

37. Inagaki N, Onouchi H, Maezawa H, Kuroda S, Kaku K. Once-weekly trelagliptin versus daily alogliptin in Japanese patients with type 2 diabetes: a randomised, double-blind, phase 3, non-inferiority study. Lancet Diabetes Endocrinol 2015; **3**(3): 191-197.

38. Ji L, Li L, Kuang J, Yang T, Kim DJ, Kadir AA, Huang CN, Lee D. Efficacy and safety of fixed-dose combination therapy, alogliptin plus metformin, in Asian patients with type 2 diabetes: A phase 3 trial. Diabetes Obes Metab 2017; **19**(5): 754-758.

39. Yang HK, Min K, Park SW, Chung CH, Park KS, Choi SH, Song KH, Kim DM, Lee MK, Sung YA, Baik SH, Kim IJ, Cha BS, Park JH, Ahn YB, Lee IK, Yoo SJ, Kim J, Park IeB, Park TS, Yoon KH. A randomized, placebo-controlled, double-blind, phase 3 trial to evaluate the efficacy and safety of anagliptin in drug-naïve patients with type 2 diabetes. Endocr J 2015; **62**(5): 449-462.

40. Park J, Park SW, Yoon KH, Kim SR, Ahn KJ, Lee JH, Mok JO, Chung CH, Han KA, Koh GP, Kang JG, Lee CB, Kim SH, Kwon NY, Kim DM. Efficacy and safety of evogliptin monotherapy in patients with type 2 diabetes and moderately elevated glycated haemoglobin levels after diet and exercise. Diabetes Obes Metab 2017; **19**(12): 1681-1687.

41. Yang SJ, Min KW, Gupta SK, Park JY, Shivane VK, Pitale SU, Agarwal PK, Sosale A, Gandhi P, Dharmalingam M, Mohan V, Mahesh U, Kim DM, Kim YS, Kim JA, Kim PK, Baik SH. A multicentre, multinational, randomized, placebo-controlled, double-blind, phase 3 trial to evaluate the efficacy and safety of gemigliptin (LC15-0444) in patients with type 2 diabetes. Diabetes Obes Metab 2013; **15**(5): 410-416.

42. Del Prato S, Barnett AH, Huisman H, Neubacher D, Woerle HJ, Dugi KA. Effect of linagliptin monotherapy on glycaemic control and markers of β-cell function in patients with inadequately controlled type 2 diabetes: a randomized controlled trial. Diabetes Obes Metab 2011; **13**(3): 258-267.

43. Chen Y, Ning G, Wang C, Gong Y, Patel S, Zhang C, Izumoto T, Woerle HJ, Wang W. Efficacy and safety of linagliptin monotherapy in Asian patients with inadequately controlled type 2 diabetes mellitus: A multinational, 24-week, randomized, clinical trial. J Diabetes Investig 2015; **6**(6): 692-698.

44. Wu W, Li Y, Chen X, Lin D, Xiang S, Shen F, Gu X. Effect of linagliptin on glycemic control in Chinese patients with newly-diagnosed, drug-naïve type 2 diabetes mellitus: a randomized controlled trial. Med Sci Monit 2015; **21**: 2678-2684.

45. Rosenstock J, Aguilar-Salinas C, Klein E, Nepal S, List J, Chen R; CV181-011 Study Investigators. Effect of saxagliptin monotherapy in treatment-naïve patients with type 2 diabetes. Curr Med Res Opin 2009; **25**(10): 2401-2411.

46. Frederich R, McNeill R, Berglind N, Fleming D, Chen R. The efficacy and safety of the dipeptidyl peptidase-4 inhibitor saxagliptin in treatment-naïve patients with type 2 diabetes mellitus: a randomized controlled trial. Diabetol Metab Syndr 2012; **4**(1): 36.

47. Pan CY, Yang W, Tou C, Gause-Nilsson I, Zhao J. Efficacy and safety of saxagliptin in drug-naïve Asian patients with type 2 diabetes mellitus: a randomized controlled trial. Diabetes Metab Res Rev 2012; **28**(3): 268-275.

48. Kumar KMP, Jain SM, Tou C, Schützer K-M. Saxagliptin as initial therapy in treatment-naive Indian adults with type 2 diabetes mellitus inadequately controlled with diet and exercise alone: a randomized, double-blind, placebo-controlled, phase IIIb clinical study. Int J Diabetes Dev Ctries 2014; **34**: 201-209.

49. Aschner P, Kipnes MS, Lunceford JK, Sanchez M, Mickel C, Williams-Herman DE; Sitagliptin Study 021 Group. Effect of the dipeptidyl peptidase-4 inhibitor sitagliptin as monotherapy on glycemic control in patients with type 2 diabetes. Diabetes Care 2006; **29**(12): 2632-2637.

50. Goldstein BJ, Feinglos MN, Lunceford JK, Johnson J, Williams-Herman DE; Sitagliptin 036 Study Group. Effect of initial combination therapy with sitagliptin, a dipeptidyl peptidase-4 inhibitor, and metformin on glycemic control in patients with type 2 diabetes. Diabetes Care 2007; **30**(8): 1979-1987.

51. Barzilai N, Guo H, Mahoney EM, Caporossi S, Golm GT, Langdon RB, Williams-Herman D, Kaufman KD, Amatruda JM, Goldstein BJ, Steinberg H. Efficacy and tolerability of sitagliptin monotherapy in elderly patients with type 2 diabetes: a randomized, double-blind, placebo-controlled trial. Curr Med Res Opin 2011; **27**(5): 1049-1058.

52. Ji L, Han P, Wang X, Liu J, Zheng S, Jou YM, O'Neill EA, Golm GT, Engel SS, Kaufman KD, Shankar RR. Randomized clinical trial of the safety and efficacy of sitagliptin and metformin co-administered to Chinese patients with type 2 diabetes mellitus. J Diabetes Investig 2016; **7**(5): 727-736.

53. Gantz I, Okamoto T, Ito Y, Okuyama K, O'Neill EA, Kaufman KD, Engel SS, Lai E; the Omarigliptin Study 020 Group. A randomized, placebo- and sitagliptin-controlled trial of the safety and efficacy of omarigliptin, a once-weekly dipeptidyl peptidase-4 inhibitor, in Japanese patients with type 2 diabetes. Diabetes Obes Metab 2017; **19**(11): 1602-1609.

54. Gupta S, Shaikh S, Joshi P, Bhure S, Suvarna V. Long-Term efficacy and safety of empagliflozin monotherapy in drug-naïve patients with type 2 diabetes in Indian subgroup: results from a 76-week extension trial of Phase III, double-blind, randomized study. Indian J Endocrinol Metab 2017; **21**(2): 286-292.

55. Zhao L, Sun T, Wang L. Chitosan oligosaccharide improves the therapeutic ef cacy of sitagliptin for the therapy of Chinese elderly patients with type 2 diabetes mellitus. Therapeutics and Clinical Risk Management 2017; **13**: 739-750.

56. Hong S, Park CY, Han KA, Chung CH, Ku BJ, Jang HC, Ahn CW, Lee MK, Moon MK, Son HS, Lee CB, Cho YW, Park SW. Efficacy and safety of teneligliptin, a novel dipeptidyl peptidase-4 inhibitor, in Korean patients with type 2 diabetes mellitus: a 24-week multicentre, randomized, double-blind, placebo-controlled phase III trial. Diabetes Obes Metab 2016; **18**(5): 528-532.

57. Dejager S, Razac S, Foley JE, Schweizer A. Vildagliptin in drug-naïve patients with type 2 diabetes: a 24-week, double-blind, randomized, placebo-controlled, multiple-dose study. Horm Metab Res 2007; **39**(3): 218-223.

58. Pi-Sunyer FX, Schweizer A, Mills D, Dejager S. Efficacy and tolerability of vildagliptin monotherapy in drug-naïve patients with type 2 diabetes. Diabetes Res Clin Pract 2007; **76**(1): 132-138.

59. Scherbaum WA, Schweizer A, Mari A, Nilsson PM, Lalanne G, Wang Y, Dunning BE, Foley JE. Evidence that vildagliptin attenuates deterioration of glycaemic control during 2-year treatment of patients with type 2 diabetes and mild hyperglycaemia. Diabetes Obes Metab 2008; **10**(11): 1114-1124.

60. Foley JE, Bunck MC, Möller-Goede DL, Poelma M, Nijpels G, Eekhoff EM, Schweizer A, Heine RJ, Diamant M. Beta cell function following 1 year vildagliptin or placebo treatment and after 12 week washout in drug-naive patients with type 2 diabetes and mild hyperglycaemia: a randomised controlled trial. Diabetologia 2011; **54**(8): 1985-1991.

61. Haak T, Meinicke T, Jones R, Weber S, von Eynatten M, Woerle HJ. Initial combination of linagliptin and metformin improves glycaemic control in type 2 diabetes: a randomized, double-blind, placebo-controlled study. Diabetes Obes Metab 2012; **14**(6): 565-574.

62. Hartley P, Shentu Y, Betz-Schiff P, Golm GT, Sisk CM, Engel SS, Shankar RR. Efficacy and tolerability of sitagliptin compared with glimepiride in elderly patients with type 2 diabetes mellitus and inadequate glycemic control: a randomized, double-blind, non-inferiority trial. Drugs Aging 2015; **32**(6): 469-476.

63. Mu Y, Pan C, Fan B, Hehnke U, Zhang X, Zhang X, Wang X, Liu J, Zhang Y, Du J, Ma J, Gong Y. Efficacy and safety of linagliptin/metformin single-pill combination as initial therapy in drug-naïve Asian patients with type 2 diabetes. Diabetes Res Clin Pract 2017; **124**: 48-56.

64. Williams-Herman D, Johnson J, Teng R, Golm G, Kaufman KD, Goldstein BJ, Amatruda JM. Efficacy and safety of sitagliptin and metformin as initial

combination therapy and as monotherapy over 2 years in patients with type 2 diabetes. Diabetes Obes Metab 2010; **12**(5): 442-51.

65. Russell-Jones D, Cuddihy RM, Hanefeld M, Kumar A, González JG, Chan M, Wolka AM, Boardman MK; DURATION-4 Study Group. Efficacy and safety of exenatide once weekly versus metformin, pioglitazone, and sitagliptin used as monotherapy in drug-naive patients with type 2 diabetes (DURATION-4): a 26-week double-blind study. Diabetes Care 2012; **35**(2): 252-258.

66. Schweizer A, Dejager S, Bosi E. Comparison of vildagliptin and metformin monotherapy in elderly patients with type 2 diabetes: a 24-week, double-blind, randomized trial. Diabetes Obes Metab 2009; **11**(8): 804-812.

67. 李运成. 沙格列汀对糖尿病微血管病变患者血浆 hs-CRP 和 Hcy 水平的影响. 山东医药 2014; **54**(38): 75-76.

68. 万靖，李俊立，罗秋荣，曾娇娥. 二甲双胍或沙格列汀单用及联用治疗 2 型糖尿病的疗效分析. 长江大学学报 2015; **12**(18): 1-3.

69. Li J, Liu C, Dong X, Zhang L. Saxagliptin add-on to metformin improves β cell functions in newly diagnosed patients with type 2 diabetes. China Medicine And Pharmacy 2013; **3**(21): 16-18.

70. Nauck MA, di Domenico M, Patel S, Kobe M, Toorawa R, Woerle HJ. Linagliptin and pioglitazone combination therapy versus monotherapy with linagliptin or pioglitazone: A randomised, double-blind, parallel-group, multinational clinical trial. Diab Vasc Dis Res 2016; **13**(4): 286-298.

71. Jin SM, Park SW, Yoon KH, Min KW, Song KH, Park KS, Park JY, Park IB, Chung CH, Baik SH, Choi SH, Lee HW, Lee IK, Kim DM, Lee MK. Anagliptin and sitagliptin as add-ons to metformin for patients with type 2 diabetes: a 24-week, multicentre, randomized, double-blind, active-controlled, phase III clinical trial with a 28-week extension. Diabetes Obes Metab 2015; **17**(5): 511-515.

72. Hong SM, Park CY, Hwang DM, Han KA, Lee CB, Chung CH, Yoon KH, Mok JO, Park KS, Park SW. Efficacy and safety of adding evogliptin versus sitagliptin for metformin-treated patients with type 2 diabetes: A 24-week randomized, controlled trial with open label extension. Diabetes Obes Metab 2017; **19**(5): 654-663.

73. Rhee EJ, Lee WY, Min KW, Shivane VK, Sosale AR, Jang HC, Chung CH, Nam-Goong IS, Kim JA, Kim SW; Gemigliptin Study 006 Group. Efficacy and safety of

the dipeptidyl peptidase-4 inhibitor gemigliptin compared with sitagliptin added to ongoing metformin therapy in patients with type 2 diabetes inadequately controlled with metformin alone. Diabetes Obes Metab 2013; **15**(6): 523-530.

74. Goldenberg R, Gantz I, Andryuk PJ, O'Neill EA, Kaufman KD, Lai E, Wang YN, Suryawanshi S, Engel SS. Randomized clinical trial comparing the efficacy and safety of treatment with the once-weekly dipeptidyl peptidase-4 (DPP-4) inhibitor omarigliptin or the once-daily DPP-4 inhibitor sitagliptin in patients with type 2 diabetes inadequately controlled on metformin monotherapy. Diabetes Obes Metab 2017; **19**(3): 394-400.

75. Xiaoyan C, Jing W, Xiaochun H, Yuyu T, Shunyou D, Yingyu F. Effects of vildagliptin versus saxagliptin on daily acute glucose fluctuations in Chinese patients with T2DM inadequately controlled with a combination of metformin and sulfonylurea. Curr Med Res Opin 2016; **32**(6): 1131-1136.

III. Supplementary Figures

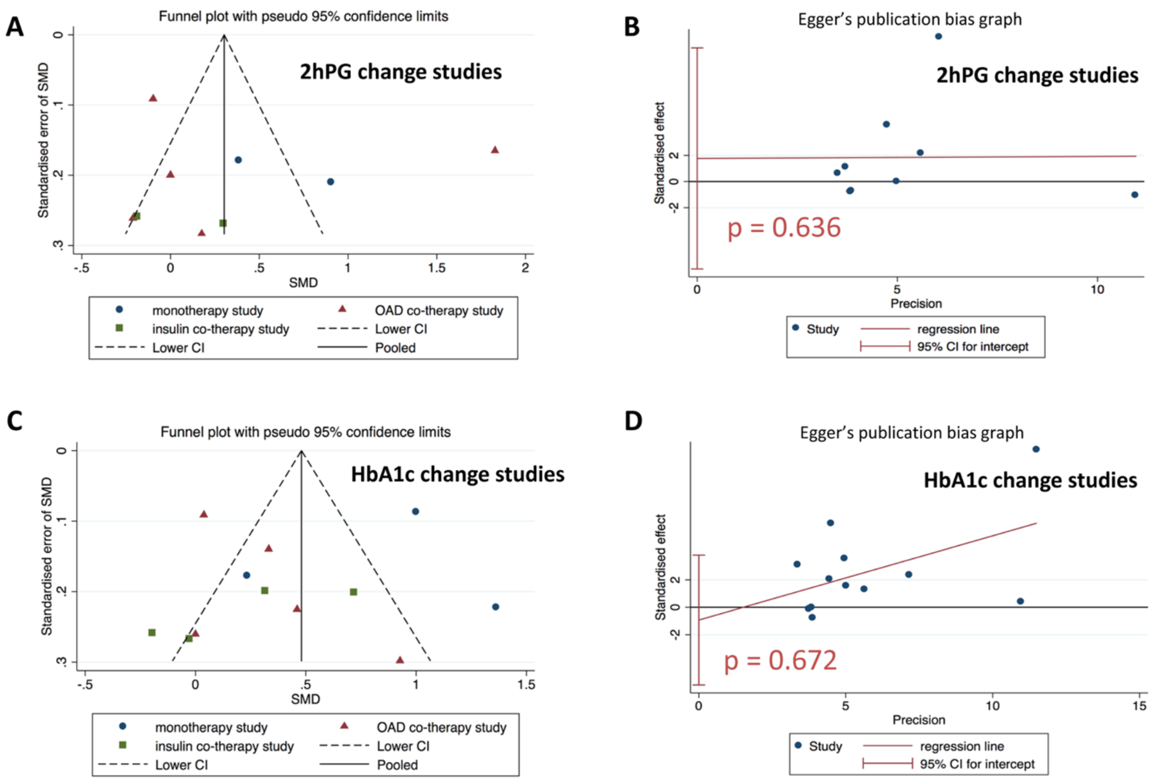

Figure S1. Funnel plots and Egger's test graphs in pair-wise meta-analysis of (**A**) and (**B**) 2-hour postprandial glucose change, (**C**) and (**D**) HbA1c change. 2hPG, 2-hour postprandial glucose; OAD, oral anti-diabetic drug.

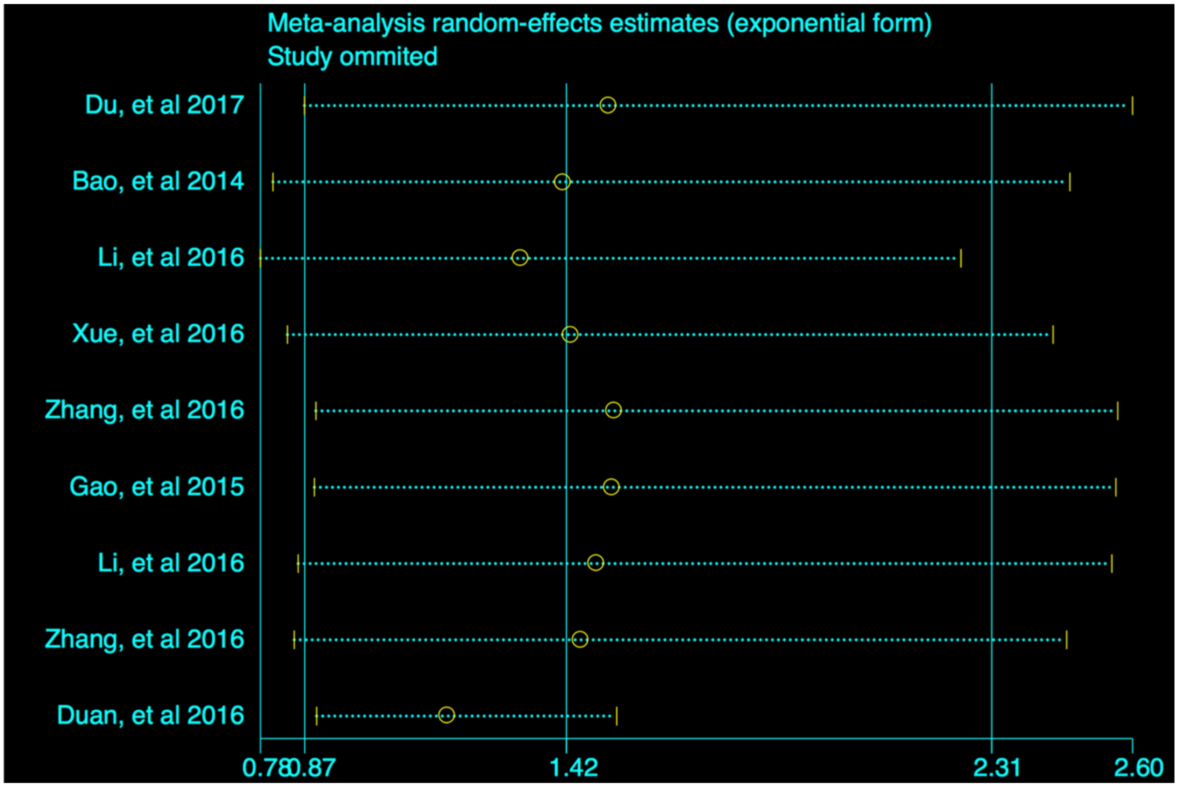

Figure S2. Sensitivity analysis of studies included in pair-wise meta-analysis of 2-hour postprandial glucose change.

A

Subgroup	Study numbers	Participant numbers: Acarbose	Participant numbers: DPP-4 inhibitors	RR of withdrawl (95% CI)	I^2, %
Monotherapy studies	3	334	554	1.26 (0.50, 3.22)	NA
OAD co-therapy studies	4	390	391	2.35 (0.46, 12.09)	76.3
Insulin co-therapy studies	4	169	168	1.00 (0.38, 2.60)	NA

p for difference among three groups = 0.422

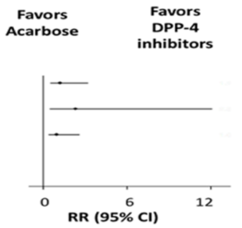

B

Subgroup	Study numbers	Participant numbers: Acarbose	Participant numbers: DPP-4 inhibitors	RR of SAE (95% CI)	I^2, %
Monotherapy studies	1	220	440	0.58 (0.12, 2.75)	NA
OAD co-therapy studies	2	333	336	1.17 (0.39, 3.47)	6.70

p for difference = 0.831

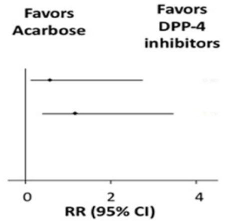

C

Subgroup	Study numbers	Participant numbers: Acarbose	Participant numbers: DPP-4 inhibitors	RR of gastrointestinal events (95% CI)	I^2, %
Monotherapy studies	1	56	440	1.86 (1.32, 2.62)	NA
OAD co-therapy studies	4	390	391	3.81 (0.93, 15.60)	3.50
Insulin co-therapy studies	2	81	81	1.00 (0.29, 3.41)	54.3

p for difference among three groups = 0.027

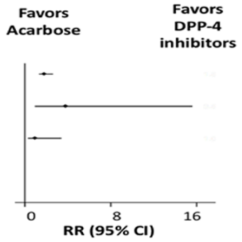

D

Subgroup	Study numbers	Participant numbers: Acarbose	Participant numbers: DPP-4 inhibitors	RR of hypoglycemia (95% CI)	I^2, %
Monotherapy studies	3	334	554	0.58 (0.17, 1.95)	32.0
OAD co-therapy studies	4	388	391	2.44 (0.83, 7.18)	56.5
Insulin co-therapy studies	3	113	113	1.31 (0.57, 2.99)	87.7

p for difference among three groups = 0.338

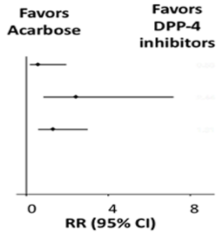

E

Subgroup	Study numbers	Participant numbers: Acarbose	Participant numbers: DPP-4 inhibitors	RR of all adverse events (95% CI)	I^2, %
Monotherapy studies	2	270	490	1.31 (1.07, 1.60)	100.0
OAD co-therapy studies	4	200	201	2.63 (1.34, 5.18)	81.4
Insulin co-therapy studies	2	81	81	1.23 (0.59, 2.58)	64.4

p for difference among three groups = 0.000

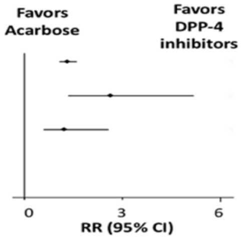

Figure S3. Pair-wise meta-analysis for comparisons between acarbose and dipeptidyl peptidase-4 inhibitors in (**A**) withdrawl, (**B**) serious adverse events, (**C**) gastrointestinal side effects, (**D**) hypoglycemia and (**E**) overall adverse events. DPP-4, dipeptidyl peptidase-4; OAD, oral anti-diabetic drugs; RR, relative risk; CI, confidence interval; SAE, serious adverse event.

A HbA1c change after over 48-week treatment

acarbose		
0.11 (-0.57, 0.67)	DPP-4 inhibitors: sitagliptin vildagliptin	
-0.26 (-0.83, 0.16)	-0.36 (-0.79, 0.01)	placebo

B

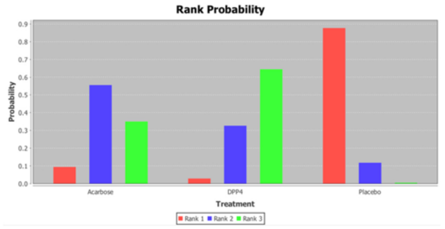

C FPG change after over 48-week treatment

acarbose		
-0.21 (-1.56, 0.78)	DPP-4 inhibitors: saxagliptin sitagliptin vildagliptin	
-0.51 (-1.57, 0.29)	-0.31 (-0.89, 0.46)	placebo

D

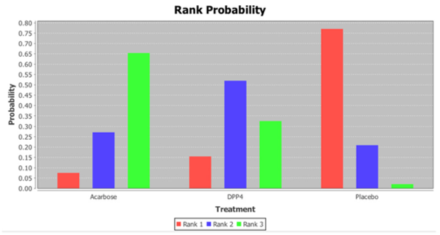

E Body weight change after over 48-week treatment

acarbose		
0.71 (-1.07, 2.48)	DPP-4 inhibitors: sitagliptin vildagliptin	
-0.07 (-1.76, 1.50)	-0.80 (-1.51, -0.12)	placebo

F

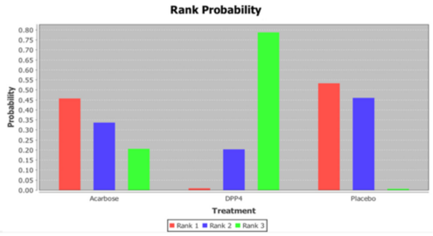

G 2hPG change in Asian participants

acarbose			
0.52 (-1.99, 3.13)	DPP-4 inhibitors: alogliptin linagliptin omarigliptin saxagliptin sitagliptin		
-1.04 (-3.17, 1.17)	-1.54 (-4.48, 1.21)	metformin	
-1.48 (-4.12, 1.29)	-2.01 (-3.66, -0.33)	-0.45 (-3.46, 2.72)	placebo

H

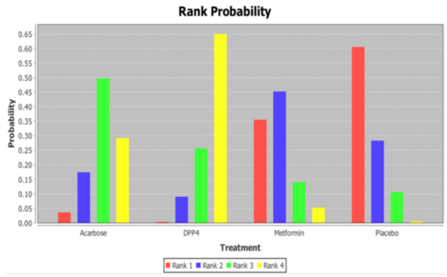

I HbA1c change in Asian participants

acarbose			
0.08 (-0.47, 0.60)	DPP-4 inhibitors: alogliptin anagliptin linagliptin omarigliptin saxagliptin sitagliptin teneligliptin vildagliptin		
0.01 (-0.72, 0.74)	-0.07 (-0.86, 0.76)	metformin	
-0.56 (-1.12, -0.05)	-0.64 (-0.97, -0.32)	-0.57 (-1.42, 0.24)	placebo

J

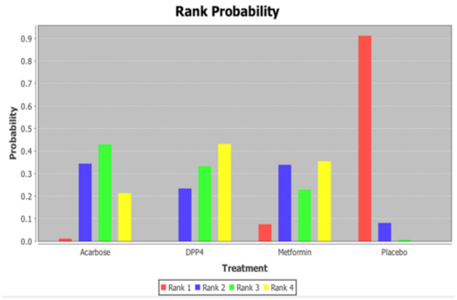

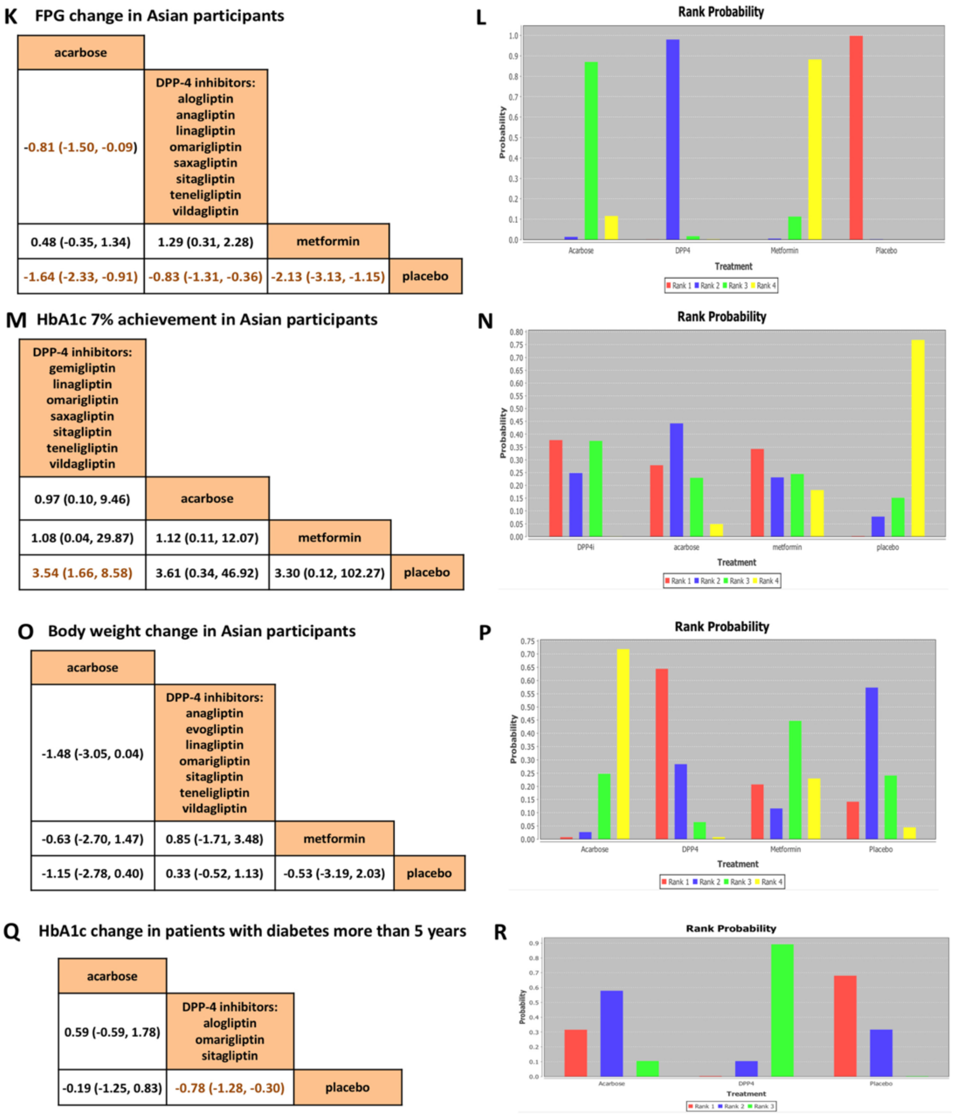

Figure S4. Subgroup analyses between acarbose and dipeptidyl peptidase-4 inhibitors in efficacy of network meta-analysis. (**A**) and (**B**) HbA1c change after more than 48-week treatment and rank probability. (**C**) and (**D**) Fasting plasma glucose change after more than 48-week treatment and rank probability. (**E**) and (**F**) Body weight change after more than 48-week treatment and rank probability. (**G**) and (**H**) 2-hour postprandial glucose change in Asian patients and rank probability. (**I**) and (**J**) HbA1c change in Asian patients and rank probability. (**K**) and (**L**) Fasting plasma glucose change in Asian patients and rank probability. (**M**) and (**N**) HbA1c < 7.0% target achievement in Asian patients and rank probability. (**O**) and (**P**) Weight change in Asian patients and rank probability. (**Q**) and (**R**) HbA1c change in patients with diabetes over 5 years and rank probability. The dark orange letters in the comparison tables indicate significant differences. DPP-4, dipeptidyl peptidase-4; 2hPG, 2-hour postprandial glucose; FPG, fasting plasma glucose.

A

**acarbose**						
-1.77 (-3.68, 0.16)	**placebo**					
-0.95 (-5.15, 3.29)	0.82 (-2.82, 4.57)	**alogliptin**				
0.27 (-3.04, 3.65)	2.04 (-0.55, 4.77)	1.20 (-3.38, 5.88)	**linagliptin**			
0.61 (-3.18, 4.29)	2.38 (-0.96, 5.76)	1.56 (-3.47, 6.55)	0.35 (-3.95, 4.54)	**omarigliptin**		
-0.46 (-4.57, 3.69)	1.29 (-2.34, 5.03)	0.49 (-4.87, 5.72)	-0.73 (-5.30, 3.78)	-1.07 (-6.05, 3.92)	**saxagliptin**	
1.10 (-1.14, 3.32)	2.88 (1.30, 4.40)	2.08 (-2.09, 5.99)	0.84 (-2.35, 3.82)	0.50 (-2.80, 3.86)	1.60 (-2.39, 5.60)	**sitagliptin**

B

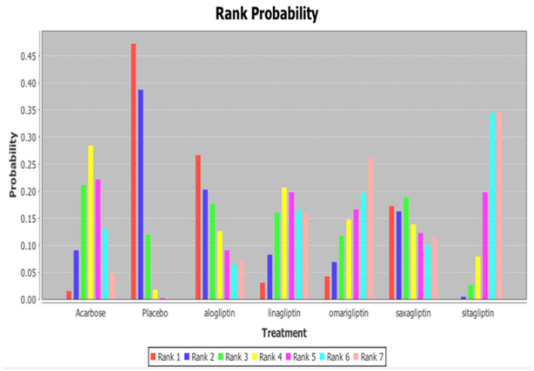

C

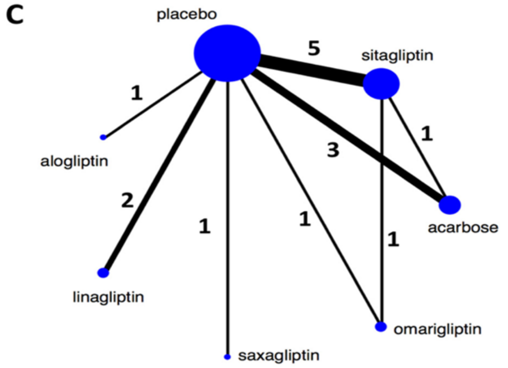

D

**acarbose**										
-0.55 (-0.83, -0.28)	**placebo**									
0.07 (-0.67, 0.79)	0.62 (-0.05, 1.31)	**alogliptin**								
-0.83 (-1.83, 0.19)	-0.28 (-1.25, 0.70)	-0.90 (-2.08, 0.31)	**anagliptin**							
0.09 (-0.48, 0.64)	0.64 (0.15, 1.11)	0.02 (-0.82, 0.85)	0.91 (-0.18, 1.99)	**linagliptin**						
0.30 (-0.58, 1.16)	0.85 (0.01, 1.68)	0.23 (-0.83, 1.32)	1.13 (-0.15, 2.43)	0.22 (-0.75, 1.17)	**omarigliptin**					
-0.10 (-0.65, 0.45)	0.45 (-0.02, 0.94)	-0.17 (-0.99, 0.68)	0.74 (-0.35, 1.81)	-0.18 (-0.84, 0.51)	-0.39 (-1.36, 0.58)	**saxagliptin**				
0.36 (-0.06, 0.78)	0.91 (0.57, 1.27)	0.29 (-0.48, 1.08)	1.19 (0.14, 2.24)	0.27 (-0.31, 0.88)	0.06 (-0.76, 0.90)	0.46 (-0.13, 1.06)	**sitagliptin**			
0.35 (-0.67, 1.36)	0.91 (-0.07, 1.88)	0.29 (-0.91, 1.44)	1.18 (-0.19, 2.55)	0.27 (-0.81, 1.37)	0.05 (-1.23, 1.33)	0.45 (-0.64, 1.53)	-0.00 (-1.05, 1.01)	**teneligliptin**		
0.00 (-0.98, 0.98)	0.55 (-0.38, 1.50)	-0.06 (-1.24, 1.10)	0.83 (-0.52, 2.17)	-0.09 (-1.13, 0.97)	-0.30 (-1.58, 0.98)	0.09 (-0.95, 1.16)	-0.36 (-1.36, 0.65)	-0.36 (-1.66, 1.01)	**trelagliptin**	
-0.02 (-0.51, 0.46)	0.53 (0.11, 0.98)	-0.09 (-0.90, 0.73)	0.81 (-0.26, 1.87)	-0.11 (-0.76, 0.56)	-0.32 (-1.25, 0.63)	0.08 (-0.58, 0.72)	-0.38 (-0.94, 0.17)	-0.37 (-1.44, 0.70)	-0.03 (-1.04, 1.01)	**vildagliptin**

E

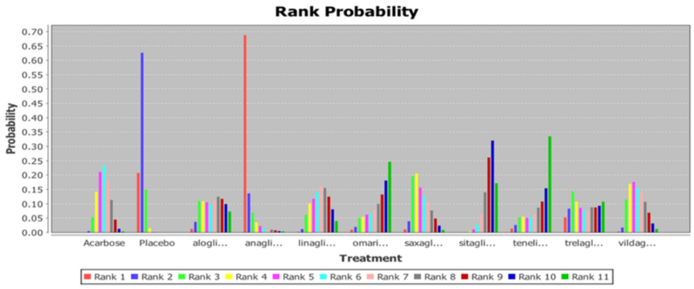

F

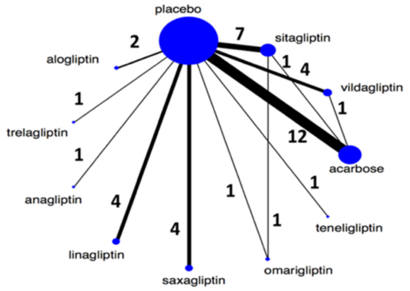

G

acarbose										
-1.14 (-1.78, -0.48)	placebo									
0.39 (-1.53, 2.38)	1.54 (-0.27, 3.40)	alogliptin								
0.35 (-1.59, 2.31)	1.49 (-0.37, 3.32)	-0.04 (-2.63, 2.50)	anagliptin							
-0.10 (-1.33, 1.15)	1.04 (0.00, 2.08)	-0.49 (-2.63, 1.62)	-0.44 (-2.49, 1.66)	linagliptin						
-0.40 (-2.08, 1.30)	0.74 (-0.82, 2.32)	-0.79 (-3.20, 1.65)	-0.75 (-3.17, 1.69)	-0.30 (-2.21, 1.59)	omarigliptin					
-0.47 (-1.57, 0.67)	0.66 (-0.25, 1.57)	-0.88 (-2.93, 1.13)	-0.83 (-2.87, 1.23)	-0.38 (-1.74, 1.01)	-0.09 (-1.86, 1.70)	saxagliptin				
-0.17 (-1.05, 0.75)	0.97 (0.27, 1.68)	-0.56 (-2.56, 1.42)	-0.52 (-2.48, 1.45)	-0.07 (-1.33, 1.20)	0.23 (-1.37, 1.77)	0.31 (-0.82, 1.45)	sitagliptin			
-0.03 (-1.96, 1.91)	1.10 (-0.73, 2.93)	-0.42 (-3.07, 2.11)	-0.39 (-2.97, 2.24)	0.07 (-2.04, 2.15)	0.35 (-2.10, 2.82)	0.45 (-1.62, 2.50)	0.12 (-1.85, 2.11)	teneligliptin		
-1.08 (-2.96, 0.86)	0.05 (-1.73, 1.84)	-1.48 (-4.10, 1.08)	-1.45 (-3.99, 1.15)	-0.99 (-3.09, 1.10)	-0.71 (-3.09, 1.71)	-0.62 (-2.61, 1.41)	-0.92 (-2.85, 1.02)	-1.06 (-3.59, 1.49)	trelagliptin	
-0.46 (-1.36, 0.51)	0.68 (-0.11, 1.48)	-0.84 (-2.89, 1.17)	-0.81 (-2.82, 1.21)	-0.36 (-1.66, 0.95)	-0.07 (-1.82, 1.69)	0.03 (-1.19, 1.24)	-0.29 (-1.33, 0.76)	-0.42 (-2.39, 1.63)	0.63 (-1.38, 2.63)	vildagliptin

H

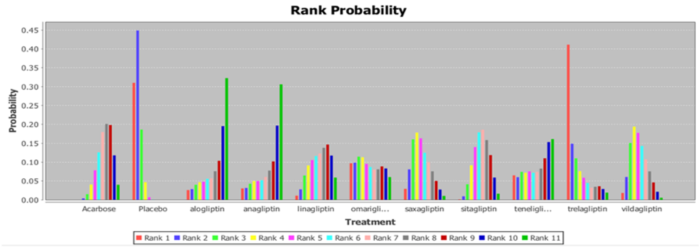

I

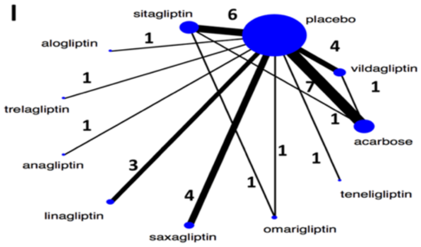

J

acarbose									
2.01 (0.41, 9.93)	alogliptin								
1.24 (0.22, 7.69)	0.63 (0.17, 2.29)	gemigliptin							
2.35 (0.50, 13.20)	1.18 (0.44, 3.66)	1.87 (0.52, 7.35)	linagliptin						
0.36 (0.05, 2.26)	0.18 (0.04, 0.71)	0.28 (0.05, 1.38)	0.15 (0.03, 0.60)	omarigliptin					
4.23 (1.05, 17.86)	2.10 (1.06, 4.39)	3.37 (1.16, 9.83)	1.80 (0.78, 3.79)	11.78 (3.70, 42.19)	placebo				
2.67 (0.62, 12.75)	1.33 (0.58, 3.47)	2.13 (0.67, 7.44)	1.13 (0.45, 2.87)	7.44 (2.15, 30.61)	0.63 (0.39, 1.11)	saxagliptin			
1.50 (0.34, 6.82)	0.74 (0.32, 1.79)	1.20 (0.36, 3.86)	0.64 (0.24, 1.53)	4.18 (1.17, 16.45)	0.35 (0.21, 0.58)	0.56 (0.26, 1.09)	sitagliptin		
0.46 (0.07, 2.99)	0.23 (0.06, 0.93)	0.37 (0.07, 1.82)	0.20 (0.05, 0.78)	1.29 (0.24, 7.33)	0.11 (0.03, 0.35)	0.17 (0.05, 0.60)	0.31 (0.08, 1.15)	teneligliptin	
1.02 (0.41, 2.56)	0.51 (0.14, 1.89)	0.82 (0.17, 3.80)	0.43 (0.10, 1.60)	2.84 (0.57, 15.30)	0.24 (0.08, 0.72)	0.38 (0.11, 1.23)	0.68 (0.20, 2.29)	2.21 (0.42, 11.11)	vildagliptin

K

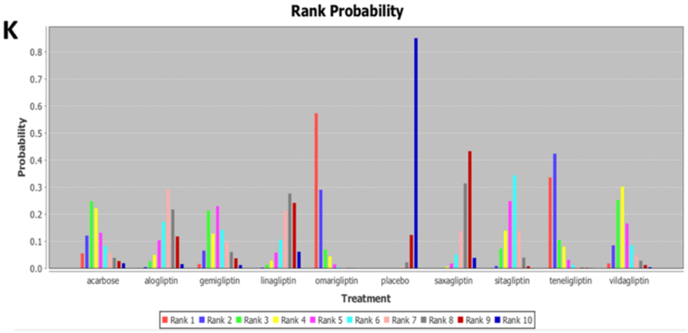

L

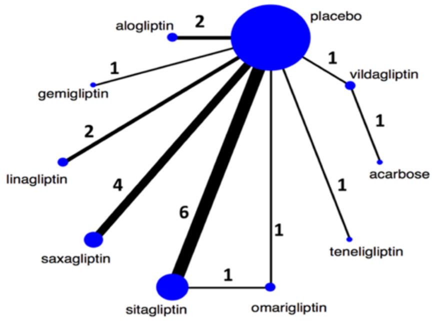

M

acarbose									
-0.95 (-2.32, 0.50)	placebo								
-0.57 (-3.30, 2.27)	0.39 (-2.03, 2.82)	alogliptin							
-0.54 (-3.41, 2.38)	0.40 (-2.12, 2.87)	0.01 (-3.52, 3.49)	anagliptin						
-1.49 (-4.19, 1.27)	-0.53 (-2.88, 1.79)	-0.93 (-4.31, 2.52)	-0.94 (-4.37, 2.56)	evogliptin					
-1.42 (-6.68, 3.92)	-0.49 (-5.61, 4.74)	-0.91 (-6.55, 4.93)	-0.86 (-6.60, 4.80)	0.06 (-5.59, 5.69)	linagliptin				
-1.57 (-3.94, 0.93)	-0.60 (-2.57, 1.45)	-1.00 (-4.14, 2.17)	-0.99 (-4.22, 2.27)	-0.06 (-3.18, 2.98)	-0.12 (-5.58, 5.44)	omarigliptin			
-1.39 (-3.27, 0.57)	-0.43 (-1.74, 0.93)	-0.82 (-3.58, 1.94)	-0.85 (-3.64, 2.06)	0.11 (-2.55, 2.79)	0.07 (-5.28, 5.37)	0.17 (-1.87, 2.18)	sitagliptin		
-1.97 (-4.60, 0.88)	-0.99 (-3.33, 1.35)	-1.41 (-4.68, 1.92)	-1.43 (-4.84, 2.07)	-0.48 (-3.75, 2.83)	-0.52 (-6.24, 5.10)	-0.38 (-3.54, 2.72)	-0.56 (-3.30, 2.10)	teneligliptin	
-1.16 (-2.73, 0.35)	-0.21 (-1.52, 1.01)	-0.58 (-3.39, 2.07)	-0.61 (-3.44, 2.22)	0.33 (-2.37, 2.91)	0.28 (-5.08, 5.52)	0.41 (-2.05, 2.66)	0.23 (-1.66, 1.98)	0.80 (-1.87, 3.37)	vildagliptin

N

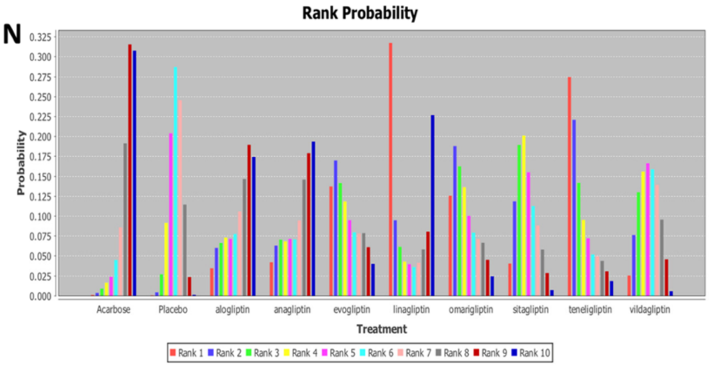

O

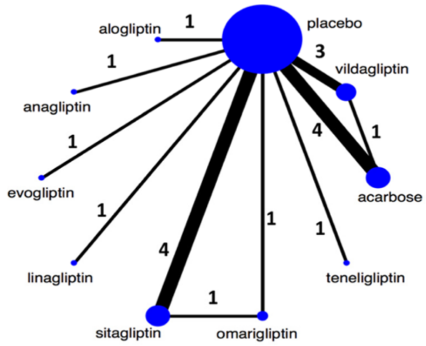

Figure S5. Network meta-analysis among acarbose, individual dipeptidyl peptidase-4 inhibitors, and placebo in (**A-C**) 2-hour postprandial glucose change, (**D-F**) HbA1c change, (**G-I**) fasting plasma glucose change, (**J-L**) relative risk of HbA1c < 7.0% target achievement, and (**M-O**) body weight change. The dark orange letters in the comparison tables suggest significant differences. In the network plots, the size of the nodes corresponds to the number of participants assigned to each treatment. Numbers by the lines indicate the cumulative number of enrolled studies for each direct comparison.

Fig 3B

acarbose	-1.08 (-1.44, -0.72)	0.19 (-0.19, 0.57)	NA	-0.73 (-1.07, -0.40)
0.96 (-0.56, 2.54)	DPP-4 inhibitors	1.27 (0.83, 1.71)	NA	0.35 (0.03, 0.67)
-0.92 (-2.56, 0.74)	-1.89 (-3.91, 0.05)	metformin	NA	-0.92 (-1.33, -0.52)
2.70 (-0.97, 6.43)	1.72 (-1.63, 5.11)	3.61 (-0.25, 7.53)	pioglitazone	NA
-1.41 (-2.85, 0.07)	-2.37 (-3.36, -1.42)	-0.49 (-2.51, 1.49)	-4.10 (-7.68, -0.66)	placebo

	N	mean	SD
acarbose	698	13.5347	3.0807
DPP-4i	1285	14.6164	4.327
placebo	1211	14.2672	3.8945
metformin	465	13.3457	3.5185

Studies in NMA: n = 18

Studies in baseline comparison: n = 16

Fig 3E

acarbose	0.05 (-0.03, 0.12)	0.42 (0.27, 0.56)	-0.03 (-0.46, -0.14)	0.04 (-0.04, 0.13)
0.05 (-0.25, 0.33)	DPP-4 inhibitors	0.37 (0.28, 0.46)	-0.35 (-0.45, -0.25)	-0.00 (-0.06, 0.05)
0.04 (-0.48, 0.55)	-0.01 (-0.52, 0.50)	metformin	-0.72 (-0.87, -0.57)	-0.38 (-0.48, -0.27)
0.58 (-0.03, 1.17)	0.52 (-0.04, 1.09)	0.54 (-0.22, 1.31)	pioglitazone	0.35 (0.22, 0.47)
-0.57 (-0.84, -0.32)	-0.62 (-0.80, -0.44)	-0.61 (-1.14, -0.09)	-1.15 (-1.74, -0.57)	placebo

	N	mean	SD
acarbose	1387	8.2123	1.5769
DPP-4i	3850	8.1657	1.0009
placebo	2909	8.1695	1.2266
metformin	599	7.7924	1.2261
pioglitazone	430	8.5156	1.163

Studies in NMA: n = 45

Studies in baseline comparison: n = 43

Fig 3H

acarbose	-0.44 (-0.60, -0.28)	0.20 (-0.01, 0.41)	-0.57 (-0.86, -0.27)	-0.32 (-0.50, -0.15)
-0.27 (-0.76, 0.24)	DPP-4 inhibitors	0.64 (0.45, 0.83)	-0.13 (-0.41, 0.15)	0.11 (-0.02, 0.24)
0.53 (-0.17, 1.23)	0.80 (0.08, 1.52)	metformin	-0.77 (-1.06, -0.48)	-0.53 (-0.74, -0.31)
1.38 (0.44, 2.33)	1.65 (0.76, 2.54)	0.85 (-0.24, 1.97)	pioglitazone	0.24 (-0.07, 0.55)
-1.15 (-1.63, -0.66)	-0.88 (-1.19, -0.57)	-1.68 (-2.43, -0.92)	-2.54 (-3.45, -1.59)	placebo

	N	mean	SD
acarbose	1095	9.0048	2.2465
DPP-4i	3437	9.4417	2.3705
placebo	2407	9.3293	2.567
metformin	631	8.8023	1.7822
pioglitazone	301	9.5708	2.6337

Studies in NMA: n = 37

Studies in baseline comparison: n = 35

Fig 3K

DPP-4 inhibitors	-0.14 (-0.22, -0.05)	0.63 (0.54, 0.72)	-0.26 (-0.36, -0.16)	0.02 (-0.04, 0.07)
1.33 (0.51, 3.64)	acarbose	0.50 (0.36, 0.64)	-0.39 (-0.55, -0.24)	-0.12 (-0.21, -0.02)
0.96 (0.31, 3.11)	0.72 (0.23, 2.28)	metformin	-0.89 (-1.03, -0.75)	-0.61 (-0.71, -0.52)
0.97 (0.39, 2.45)	0.73 (0.25, 2.14)	1.01 (0.26, 3.86)	pioglitazone	0.28 (0.17, 0.38)
2.70 (1.86, 4.01)	2.04 (0.70, 5.74)	2.82 (0.81, 9.05)	2.78 (1.03, 7.55)	placebo

	N	mean	SD
acarbose	717	8.1227	1.3641
DPP-4i	3009	8.2566	0.9568
placebo	1799	8.2387	0.9187
metformin	517	7.6253	1.0618
pioglitazone	430	8.5156	1.163

Studies in NMA: n = 23

Studies in baseline comparison: n = 21

Fig 3N

acarbose	-4.93 (-6.61, -3.26)	-0.32 (-1.75, 1.12)	-15.72 (-17.94, -13.48)	-2.57 (-4.18, -0.96)
-1.23 (-2.08, -0.33)	DPP-4 inhibitors	4.62 (2.62, 6.61)	-10.78 (-13.77, -7.80)	2.36 (0.67, 4.06)
-0.52 (-1.75, 0.70)	0.69 (-0.53, 1.89)	metformin	-15.4 (-17.88, -12.92)	-2.25 (-4.16, -0.35)
-3.42 (-4.75, -2.05)	-2.22 (-3.49, -0.91)	-2.91 (-4.59, -1.21)	pioglitazone	13.15 (10.29, 16.00)
-1.00 (-1.86, -0.09)	0.22 (-0.28, 0.75)	-0.48 (-1.73, 0.80)	2.43 (1.05, 3.82)	placebo

	N	mean	SD
acarbose	533	70.3841	10.6874
DPP-4i	863	75.3171	17.8035
placebo	742	72.9548	16.6121
metformin	351	70.7	10.6
pioglitazone	163	86.1	17.8

Studies in NMA: n = 21

Studies in baseline comparison: n = 15

Fig S4A. **HbA1c change after over 48-week treatment**

acarbose	0.31 (0.01, 0.60)	0.22 (-0.05, 0.50)
0.11 (-0.57, 0.67)	DPP-4 inhibitors	-0.08 (-0.31, 0.15)
-0.26 (-0.83, 0.16)	-0.36 (-0.79, 0.01)	placebo

	N	mean	SD
acarbose	187	7.5837	1.7906
DPP-4i	198	7.2785	1.1073
placebo	387	7.3601	1.4474

Studies in NMA: n = 7

Studies in baseline comparison: n = 6

Fig S4C. **FPG change after over 48-week treatment**

acarbose	-0.35 (-0.76, 0.05)	-0.33 (-0.71, 0.06)
-0.21 (-1.56, 0.78)	DPP-4 inhibitors	0.03 (-0.31, 0.36)
-0.51 (-1.57, 0.29)	-0.31 (-0.89, 0.46)	placebo

	N	mean	SD
acarbose	145	7.4255	1.9679
DPP-4i	198	7.7797	1.8233
placebo	343	7.7529	1.9833

Studies in NMA: n = 6

Studies in baseline comparison: n = 6

Fig S4E. **Body weight change after over 48-week treatment**

acarbose	5.82 (-0.16, 11.80)	2.3 (-2.04, 6.64)
0.71 (-1.07, 2.48)	DPP-4 inhibitors	-3.52 (-9.23, 2.19)
-0.07 (-1.76, 1.50)	-0.80 (-1.51, -0.12)	placebo

	N	mean	SD
acarbose	36	74.3	8.6
DPP-4i	27	68.48	14.98
placebo	66	72	11.47

Studies in NMA: n = 3

Studies in baseline comparison: n = 2

Fig S4G. **2hPG change in Asian participants**

acarbose	-1.13 (-1.54, -0.71)	-0.17 (-0.58, 0.24)	-1.19 (-1.65, -0.72)
0.52 (-1.99, 3.13)	DPP-4 inhibitors	0.95 (0.49, 1.41)	-0.06 (-0.58, 0.46)
-1.04 (-3.17, 1.17)	-1.54 (-4.48, 1.21)	metformin	-1.01 (-1.53, -0.50)
-1.48 (-4.12, 1.29)	-2.01 (-3.66, -0.33)	-0.45 (-3.46, 2.72)	placebo

	N	mean	SD
acarbose	553	13.1719	3.1376
DPP-4i	519	14.2976	3.7741
placebo	363	14.3593	3.9806
metformin	465	13.3457	3.5185

Studies in NMA: n = 19

Studies in baseline comparison: n = 19

Fig S4I. **HbA1c change in Asian participants**

acarbose	-0.04 (-0.14, 0.05)	0.35 (0.17, 0.52)	-0.16 (-0.28, -0.04)
0.08 (-0.47, 0.60)	DPP-4 inhibitors	0.39 (0.28, 0.50)	-0.11 (-0.19, -0.04)
0.01 (-0.72, 0.74)	-0.07 (-0.86, 0.76)	metformin	-0.50 (-0.64, -0.36)
-0.56 (-1.12, -0.05)	-0.64 (-0.97, -0.32)	-0.57 (-1.42, 0.24)	placebo

	N	mean	SD
acarbose	847	8.1747	1.5604
DPP-4i	1922	8.2155	0.9432
placebo	1276	8.3301	1.2453
metformin	433	7.8278	1.3925

Studies in NMA: n = 20

Studies in baseline comparison: n = 20

Fig S4K. **FPG change in Asian participants**

acarbose	-0.17 (-0.35, 0.01)	0.46 (0.24, 0.68)	0.11 (-0.08, 0.31)
-0.81 (-1.50, -0.09)	DPP-4 inhibitors	0.63 (0.42, 0.85)	0.28 (0.11, 0.46)
0.48 (-0.35, 1.34)	1.29 (0.31, 2.28)	metformin	-0.35 (-0.58, -0.12)
-1.64 (-2.33, -0.91)	-0.83 (-1.31, -0.36)	-2.13 (-3.13, -1.15)	placebo

	N	mean	SD
acarbose	875	9.1219	2.0876
DPP-4i	1601	9.2943	2.1786
placebo	1058	9.0094	2.2753
metformin	465	8.6603	1.5858

Studies in NMA: n = 11
Studies in baseline comparison: n = 10

Fig S4M. **HbA1c 7% achievement in Asian participants**

DPP-4 inhibitors	-0.36 (-0.46, -0.26)	-0.33 (-0.44, -0.22)	0.03 (-0.04, 0.10)
0.97 (0.10, 9.46)	acarbose	0.32 (0.15, 0.49)	-0.33 (-0.44, -0.22)
1.08 (0.04, 29.87)	1.12 (0.11, 12.07)	metformin	-0.65 (-0.77, -0.53)
3.54 (1.66, 8.58)	3.61 (0.34, 46.92)	3.30 (0.12, 102.27)	placebo

	N	mean	SD
acarbose	581	7.9103	1.2858
DPP-4i	1606	8.2689	0.8989
placebo	1028	8.2387	0.894
metformin	351	7.59	1.22

Studies in NMA: n = 11
Studies in baseline comparison: n = 10

Fig S4O. **Body weight change in Asian participants**

acarbose	-0.21 (-1.74, 1.32)	-1.49 (-3.00, 0.01)	0.24 (-1.34, 1.82)
-1.48 (-3.05, 0.04)	DPP-4 inhibitors	-1.28 (-2.91, 0.36)	0.45 (-1.12, 2.02)
-0.63 (-2.70, 1.47)	0.85 (-1.71, 3.48)	metformin	1.73 (0.04, 3.42)
-1.15 (-2.78, 0.40)	0.33 (-0.52, 1.13)	-0.53 (-3.19, 2.03)	placebo

	N	mean	SD
acarbose	424	69.2085	10.6326
DPP-4i	609	69.4217	13.3746
placebo	536	68.9677	13.6587
metformin	351	70.7	10.6

Studies in NMA: n = 11
Studies in baseline comparison: n = 10

Fig S4Q. **HbA1c change in patients with diabetes more than 5 years**

acarbose	NA	NA
0.59 (-0.59, 1.78)	DPP-4 inhibitors	-0.04 (-0.15, 0.08)
-0.19 (-1.25, 0.83)	-0.78 (-1.28, -0.30)	placebo

	N	mean	SD
DPP-4i	419	7.7989	0.767
placebo	287	7.838	0.7682

Studies in NMA: n = 5
Studies in baseline comparison: n = 4

Fig S5A.

acarbose	—	—	—	—	—	—
-1.77 (-3.68, 0.16)	placebo	—	—	—	—	-0.84 (-1.23, -0.45)
-0.95 (-5.15, 3.29)	0.82 (-2.82, 4.57)	alogliptin	—	—	—	—
0.27 (-3.04, 3.65)	2.04 (-0.55, 4.77)	1.20 (-3.38, 5.88)	linagliptin	—	—	—
0.61 (-3.18, 4.29)	2.38 (-0.96, 5.76)	1.56 (-3.47, 6.55)	0.35 (-3.95, 4.54)	omarigliptin	—	—
-0.46 (-4.57, 3.69)	1.29 (-2.34, 5.03)	0.49 (-4.87, 5.72)	-0.73 (-5.30, 3.78)	-1.07 (-6.05, 3.92)	saxagliptin	—
1.10 (-1.14, 3.32)	2.88 (1.30, 4.40)	2.08 (-2.09, 5.99)	0.84 (-2.35, 3.82)	0.50 (-2.80, 3.86)	1.60 (-2.39, 5.60)	sitagliptin

	N	mean	SD
acarbose	275	14.437	2.3736
placebo	1211	14.2672	3.8945
linagliptin	370	13.9361	4.0785
omarigliptin	166	13.4	3.5
sitagliptin	701	15.1072	4.5465
trelagliptin	101	13.32	2.83

Studies in NMA: n = 13
Studies in baseline comparison: n = 12

Fig S5D.

acarbose	0.25 (0.15, 0.35)	—	—	—	—	—	—	—	—	—
-0.55 (-0.83, -0.28)	placebo	—	—	-0.05 (-0.14, 0.05)	-0.27 (-0.46, -0.08)	—	0.13 (0.04, 0.22)	—	—	0.07 (-0.02, 0.17)
0.07 (-0.67, 0.79)	0.62 (-0.05, 1.31)	alogliptin	—	—	—	—	—	—	—	—
-0.83 (-1.83, 0.19)	-0.28 (-1.25, 0.70)	-0.90 (-2.08, 0.31)	anagliptin	—	—	—	-1.11 (-1.46, -0.76)	—	—	—
0.09 (-0.48, 0.64)	0.64 (0.15, 1.11)	0.02 (-0.82, 0.85)	0.91 (-0.18, 1.99)	linagliptin	—	—	—	—	—	—
0.30 (-0.58, 1.16)	0.85 (0.01, 1.68)	0.23 (-0.83, 1.32)	1.13 (-0.15, 2.43)	0.22 (-0.75, 1.17)	omarigliptin	—	—	—	—	—
-0.10 (-0.65, 0.45)	0.45 (-0.02, 0.94)	-0.17 (-0.99, 0.68)	0.74 (-0.35, 1.81)	-0.18 (-0.84, 0.51)	-0.39 (-1.36, 0.58)	saxagliptin	—	—	—	—
0.36 (-0.06, 0.78)	0.91 (0.57, 1.27)	0.29 (-0.48, 1.08)	1.19 (0.14, 2.24)	0.27 (-0.31, 0.88)	0.06 (-0.76, 0.90)	0.46 (-0.13, 1.06)	sitagliptin	—	—	—
0.35 (-0.67, 1.36)	0.91 (-0.07, 1.88)	0.29 (-0.91, 1.44)	1.18 (-0.19, 2.55)	0.27 (-0.81, 1.37)	0.05 (-1.23, 1.33)	0.45 (-0.64, 1.53)	-0.00 (-1.05, 1.01)	teneligliptin	—	—
0.00 (-0.98, 0.98)	0.55 (-0.38, 1.50)	-0.06 (-1.24, 1.10)	0.83 (-0.52, 2.17)	-0.09 (-1.13, 0.97)	-0.30 (-1.58, 0.98)	0.09 (-0.95, 1.16)	-0.36 (-1.36, 0.65)	-0.36 (-1.66, 1.01)	trelagliptin	—
-0.02 (-0.51, 0.46)	0.53 (0.11, 0.98)	-0.09 (-0.90, 0.73)	0.81 (-0.26, 1.87)	-0.11 (-0.76, 0.56)	-0.32 (-1.25, 0.63)	0.08 (-0.58, 0.72)	-0.38 (-0.94, 0.17)	-0.37 (-1.44, 0.70)	-0.03 (-1.04, 1.01)	vildagliptin

	N	mean	SD
acarbose	856	8.4172	1.6358
placebo	2909	8.1695	1.2266
alogliptin	163	8.48	0.71
anagliptin	33	7.19	0.73
linagliptin	712	8.1241	0.9565
omarigliptin	166	7.9	0.7
saxagliptin	563	8.1075	0.8762
sitagliptin	778	8.2996	1.0106
teneligliptin	99	7.63	0.69
trelagliptin	101	7.73	0.85
vildagliptin	720	8.2429	1.1088

Studies in NMA: n = 38

Studies in baseline comparison: n = 36

Fig S5G.

acarbose	0.08 (-0.14, 0.30)	—	—	—	—	—	—	—	—	—
-1.14 (-1.78, -0.48)	placebo	—	—	0.18 (-0.06, 0.42)	—	—	0.73 (0.52, 0.95)	—	—	—
0.39 (-1.53, 2.38)	1.54 (-0.27, 3.40)	alogliptin	—	—	—	—	—	—	—	—
0.35 (-1.59, 2.31)	1.49 (-0.37, 3.32)	-0.04 (-2.63, 2.50)	anagliptin	—	—	—	—	—	—	—
-0.10 (-1.33, 1.15)	1.04 (0.00, 2.08)	-0.49 (-2.63, 1.62)	-0.44 (-2.49, 1.66)	linagliptin	—	—	—	—	—	—
-0.40 (-2.08, 1.30)	0.74 (-0.82, 2.32)	-0.79 (-3.20, 1.65)	-0.75 (-3.17, 1.69)	-0.30 (-2.21, 1.59)	omarigliptin	—	—	—	—	—
-0.47 (-1.57, 0.67)	0.66 (-0.25, 1.57)	-0.88 (-2.93, 1.13)	-0.83 (-2.87, 1.23)	-0.38 (-1.74, 1.01)	-0.09 (-1.86, 1.70)	saxagliptin	—	—	—	—
-0.17 (-1.05, 0.75)	0.97 (0.27, 1.68)	-0.56 (-2.56, 1.42)	-0.52 (-2.48, 1.45)	-0.07 (-1.33, 1.20)	0.23 (-1.37, 1.77)	0.31 (-0.82, 1.45)	sitagliptin	—	—	—
-0.03 (-1.96, 1.91)	1.10 (-0.73, 2.93)	-0.42 (-3.07, 2.11)	-0.39 (-2.97, 2.24)	0.07 (-2.04, 2.15)	0.35 (-2.10, 2.82)	0.45 (-1.62, 2.50)	0.12 (-1.85, 2.11)	teneligliptin	—	—
-1.08 (-2.96, 0.86)	0.05 (-1.73, 1.84)	-1.48 (-4.10, 1.08)	-1.45 (-3.99, 1.15)	-0.99 (-3.09, 1.10)	-0.71 (-3.09, 1.71)	-0.62 (-2.61, 1.41)	-0.92 (-2.85, 1.02)	-1.06 (-3.59, 1.49)	trelagliptin	—
-0.46 (-1.36, 0.51)	0.68 (-0.11, 1.48)	-0.84 (-2.89, 1.17)	-0.81 (-2.82, 1.21)	-0.36 (-1.66, 0.95)	-0.07 (-1.82, 1.69)	0.03 (-1.19, 1.24)	-0.29 (-1.33, 0.76)	-0.42 (-2.39, 1.63)	0.63 (-1.38, 2.63)	vildagliptin

	N	mean	SD
acarbose	672	9.4078	2.4834
placebo	2407	9.3293	2.567
anagliptin	33	7.74	1.59
linagliptin	512	9.5122	2.2878
omarigliptin	166	9	1.7
saxagliptin	563	9.0339	2.1422
sitagliptin	728	10.0637	2.6148
teneligliptin	99	8.62	1.6
trelagliptin	101	8.74	1.68
vildagliptin	720	9.5768	2.544

Studies in NMA: n = 30

Studies in baseline comparison: n = 29

Fig S5J.

acarbose	—	—	—	—	0.36 (0.23, 0.49)	—	—	—	—
2.01 (0.41, 9.93)	alogliptin	—	—	—	0.24 (0.10, 0.39)	—	—	—	—
1.24 (0.22, 7.69)	0.63 (0.17, 2.29)	gemigliptin	—	—	NA	—	—	—	—
2.35 (0.50, 13.20)	1.18 (0.44, 3.66)	1.87 (0.52, 7.35)	linagliptin	—	—	—	—	—	—
0.36 (0.05, 2.26)	0.18 (0.04, 0.71)	0.28 (0.05, 1.38)	0.15 (0.03, 0.60)	omarigliptin	-0.34 (-0.48, -0.20)	—	—	—	—
4.23 (1.05, 17.86)	2.10 (1.06, 4.39)	3.37 (1.16, 9.83)	1.80 (0.78, 3.79)	11.78 (3.70, 42.19)	placebo	—	0.14 (0.06, 0.23)	-0.61 (-0.79, -0.42)	0.31 (0.22, 0.40)
2.67 (0.62, 12.75)	1.33 (0.58, 3.47)	2.13 (0.67, 7.44)	1.13 (0.45, 2.87)	7.44 (2.15, 30.61)	0.63 (0.39, 1.11)	saxagliptin	—	—	—
1.50 (0.34, 6.82)	0.74 (0.32, 1.79)	1.20 (0.36, 3.86)	0.64 (0.24, 1.53)	4.18 (1.17, 16.45)	0.35 (0.21, 0.58)	0.56 (0.26, 1.09)	sitagliptin	—	—
0.46 (0.07, 2.99)	0.23 (0.06, 0.93)	0.37 (0.07, 1.82)	0.20 (0.05, 0.78)	1.29 (0.24, 7.33)	0.11 (0.03, 0.35)	0.17 (0.05, 0.60)	0.31 (0.08, 1.15)	teneligliptin	—
1.02 (0.41, 2.56)	0.51 (0.14, 1.89)	0.82 (0.17, 3.80)	0.43 (0.10, 1.60)	2.84 (0.57, 15.30)	0.24 (0.08, 0.72)	0.38 (0.11, 1.23)	0.68 (0.20, 2.29)	2.21 (0.42, 11.11)	vildagliptin

	N	mean	SD
acarbose	220	8.6	1
placebo	1799	8.2387	0.9187
alogliptin	163	8.48	0.71
linagliptin	342	8.2614	1.0063
omarigliptin	166	7.9	0.7
saxagliptin	563	8.1075	0.8762
sitagliptin	678	8.3806	1.0277
teneligliptin	99	7.63	0.69
vildagliptin	531	8.5486	0.8902

Studies in NMA: n = 18

Studies in baseline comparison: n = 16

Fig S5M.

acarbose	-1.97 (-4.59, 0.64)	—	—	—	—	—	—	—	—
-0.95 (-2.32, 0.50)	placebo	—	—	—	—	—	—	—	—
-0.57 (-3.30, 2.27)	0.39 (-2.03, 2.82)	alogliptin	—	—	—	—	—	—	—
-0.54 (-3.41, 2.38)	0.40 (-2.12, 2.87)	0.01 (-3.52, 3.49)	anagliptin	—	—	—	—	—	—
-1.49 (-4.19, 1.27)	-0.53 (-2.88, 1.79)	-0.93 (-4.31, 2.52)	-0.94 (-4.37, 2.56)	evogliptin	—	—	—	—	—
-1.42 (-6.68, 3.92)	-0.49 (-5.61, 4.74)	-0.91 (-6.55, 4.93)	-0.86 (-6.60, 4.80)	0.06 (-5.59, 5.69)	linagliptin	—	—	—	—
-1.57 (-3.94, 0.93)	-0.60 (-2.57, 1.45)	-1.00 (-4.14, 2.17)	-0.99 (-4.22, 2.27)	-0.06 (-3.18, 2.98)	-0.12 (-5.58, 5.44)	omarigliptin	—	—	—
-1.39 (-3.27, 0.57)	-0.43 (-1.74, 0.93)	-0.82 (-3.58, 1.94)	-0.85 (-3.64, 2.06)	0.11 (-2.55, 2.79)	0.07 (-5.28, 5.37)	0.17 (-1.87, 2.18)	sitagliptin	—	—
-1.97 (-4.60, 0.88)	-0.99 (-3.33, 1.35)	-1.41 (-4.68, 1.92)	-1.43 (-4.84, 2.07)	-0.48 (-3.75, 2.83)	-0.52 (-6.24, 5.10)	-0.38 (-3.54, 2.72)	-0.56 (-3.30, 2.10)	teneligliptin	—
-1.16 (-2.73, 0.35)	-0.21 (-1.52, 1.01)	-0.58 (-3.39, 2.07)	-0.61 (-3.44, 2.22)	0.33 (-2.37, 2.91)	0.28 (-5.08, 5.52)	0.41 (-2.05, 2.66)	0.23 (-1.66, 1.98)	0.80 (-1.87, 3.37)	vildagliptin

	N	mean	SD
acarbose	172	70.9802	11.078
placebo	742	72.9548	16.6121
anagliptin	33	66.15	10.56
evogliptin	80	67.6	11.5
linagliptin	34	67.05	8.12
omarigliptin	166	67	13
sitagliptin	197	74.9744	14.7806
teneligliptin	99	65.81	11.39
vildaglipin	91	90.8	19.9

Studies in NMA: n = 17

Studies in baseline comparison: n = 13

Figure S6. The baseline comparisons for the results of network meta-analysis. The serial numbers of the colorful tables are the same figure numbers as in the manuscript text. **In the left tables**, results of the **network meta-analysis mentioned in the manuscript** are presented in the **left lower half**, and results of the **baseline comparisons** in the **upper right half** (that is, in the **light gray cells**). The baseline comparisons are calculated by a two-sided student t test (via Stata 14.0). The dark orange letters in the comparison tables indicate significant differences. **In the right tables**, the information in sample size, mean and standard deviation of each treatment across studies at baseline are displayed. **Fig 3B**, **3E**, **3H**, **3K** and **3N** are showing the network meta-analysis comparisons in the 2hPG change, HbA1c change, FPG change, HbA1c < 7% achievement, and weight change, respectively. Both **Fig S4A-Q** and **Fig S5A-M** are showing the corresponding subgroup analyses results. DPP-4, dipeptidyl peptidase-4; DPP-4i, DPP-4 inhibitor; 2hPG, 2-hour postprandial glucose; FPG, fasting plasma glucose; N, sample size; NA, not applicable; NMA, network meta-analysis; SD, standard deviation; —, not calculated (for no significant results in NMA, or for including only one study in the treatment group).

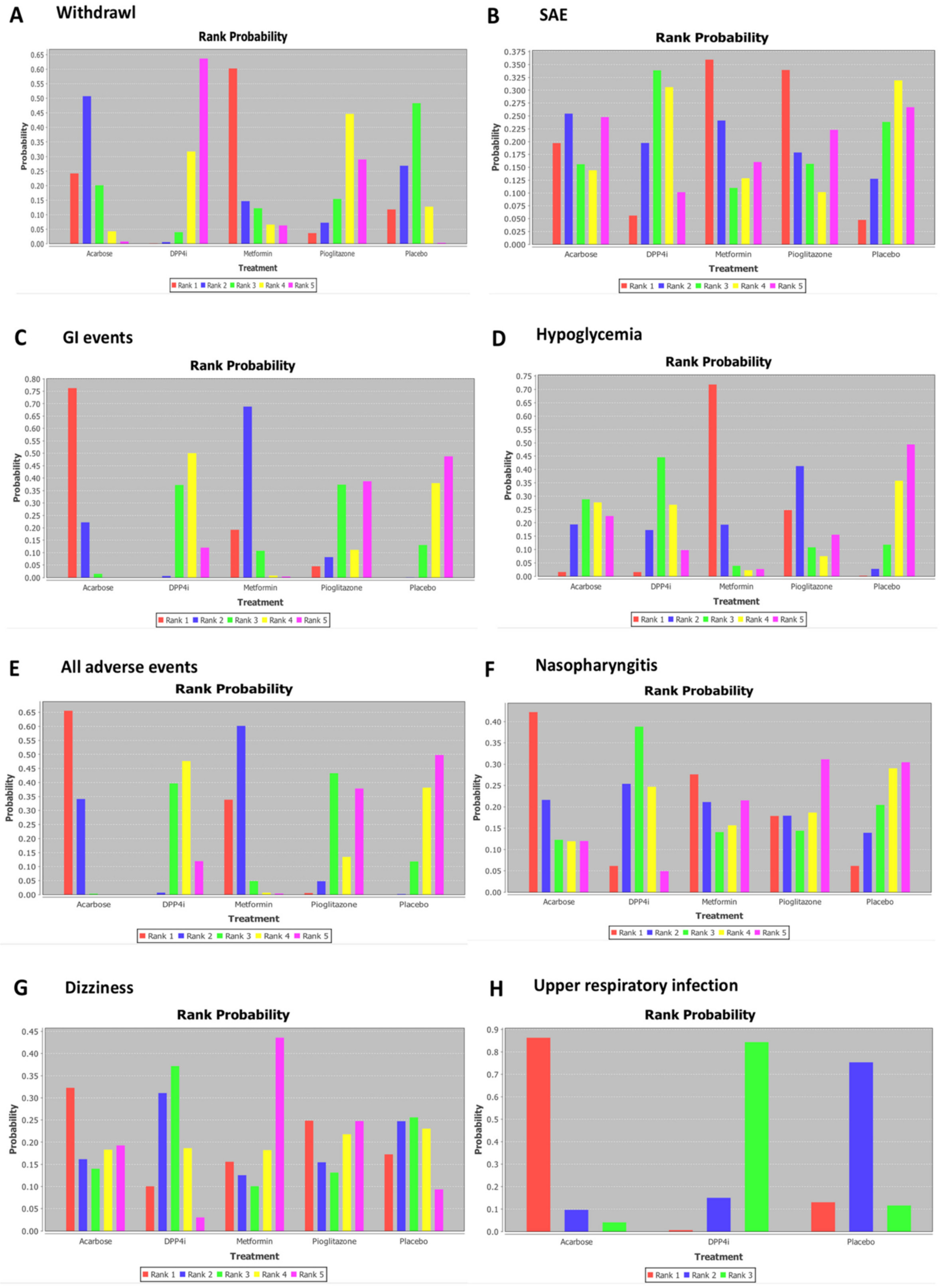
A
Withdrawl
Rank Probability
Probability
Acarbose
DPP4i
Metformin
Pioglitazone
Placebo
Treatment
Rank 1
Rank 2
Rank 3
Rank 4
Rank 5
B
SAE
Rank Probability
Probability
Acarbose
DPP4i
Metformin
Pioglitazone
Placebo
Treatment
Rank 1
Rank 2
Rank 3
Rank 4
Rank 5
C
GI events
Rank Probability
Probability
Acarbose
DPP4i
Metformin
Pioglitazone
Placebo
Treatment
Rank 1
Rank 2
Rank 3
Rank 4
Rank 5
D
Hypoglycemia
Rank Probability
Probability
Acarbose
DPP4i
Metformin
Pioglitazone
Placebo
Treatment
Rank 1
Rank 2
Rank 3
Rank 4
Rank 5
E
All adverse events
Rank Probability
Probability
Acarbose
DPP4i
Metformin
Pioglitazone
Placebo
Treatment
Rank 1
Rank 2
Rank 3
Rank 4
Rank 5
F
Nasopharyngitis
Rank Probability
Probability
Acarbose
DPP4i
Metformin
Pioglitazone
Placebo
Treatment
Rank 1
Rank 2
Rank 3
Rank 4
Rank 5
G
Dizziness
Rank Probability
Probability
Acarbose
DPP4i
Metformin
Pioglitazone
Placebo
Treatment
Rank 1
Rank 2
Rank 3
Rank 4
Rank 5
H
Upper respiratory infection
Rank Probability
Probability
Acarbose
DPP4i
Placebo
Treatment
Rank 1
Rank 2
Rank 3

Figure S7. Rank probabilities among acarbose, dipeptidyl peptidase-4 inhibitors, metformin, pioglitazone, and placebo in network meta-analysis in (**A**) withdrawl, (**B**) serious adverse events, (**C**) gastrointestinal side effects, (**D**) hypoglycemia, (**E**) overall adverse events, (**F**) nasopharyngitis, (**G**) dizziness, and (**H**) upper respiratory tract infection. DPP4i, dipeptidyl peptidase 4 inhibitor; SAE, serious adverse events; GI, gastrointestinal.

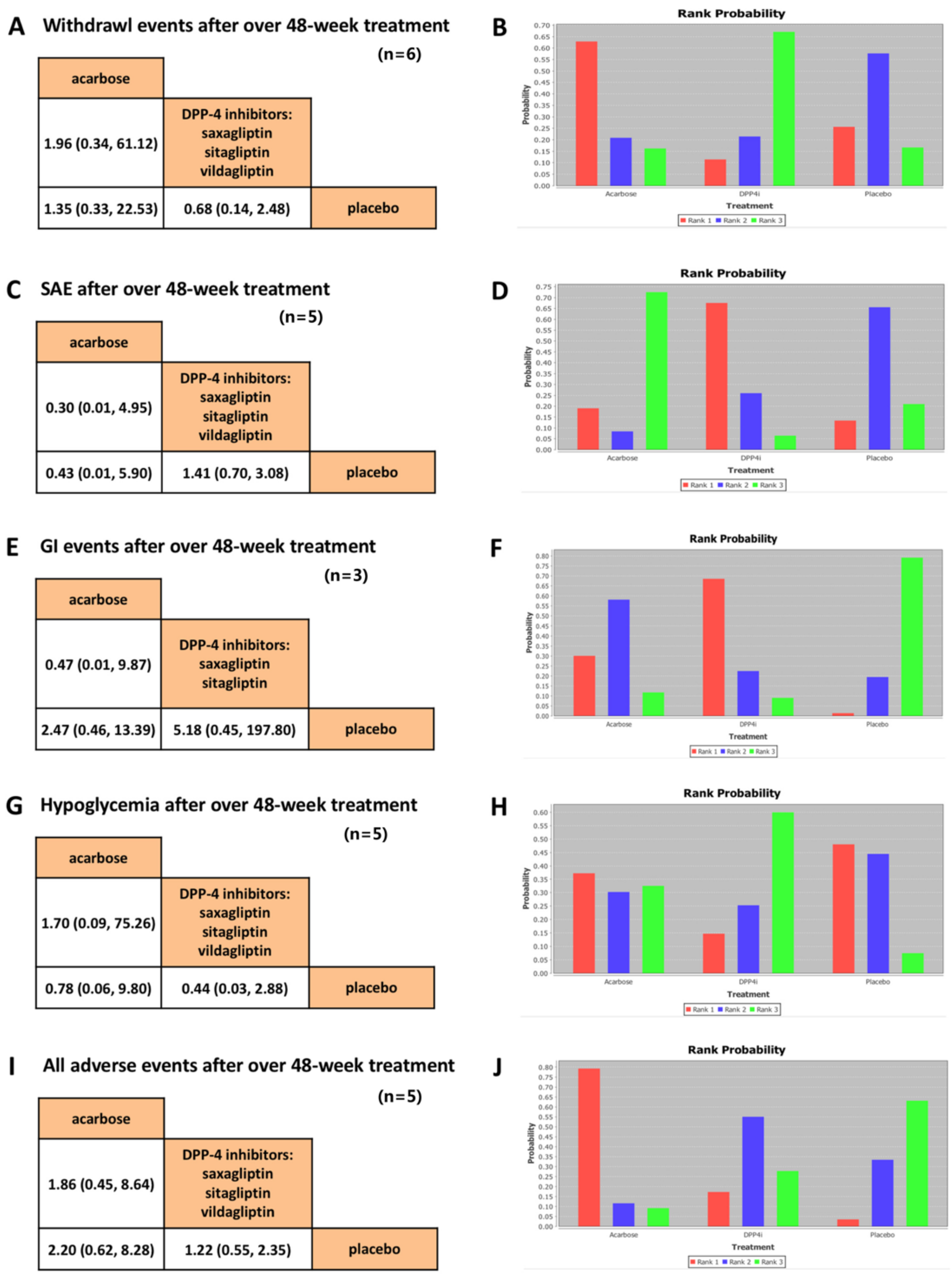

Figure S8. Subgroup analyses between acarbose and dipeptidyl peptidase-4 inhibitors in safety issues of network meta-analysis. (**A**) and (**B**) Withdrawl events after more than 48-week treatment and rank probability. (**C**) and (**D**) Serious adverse events after more than 48-week treatment and rank probability. (**E**) and (**F**) Gastrointestinal side effects after more than 48-week treatment and rank probability. (**G**) and (**H**) Hypoglycemia after more than 48-week treatment and rank probability. (**I**) and (**J**) Overall adverse events after more than 48-week treatment and rank probability. The dark orange letters in the comparison tables indicate significant differences. DPP-4, dipeptidyl peptidase-4; SAE, serious adverse events; GI, gastrointestinal.

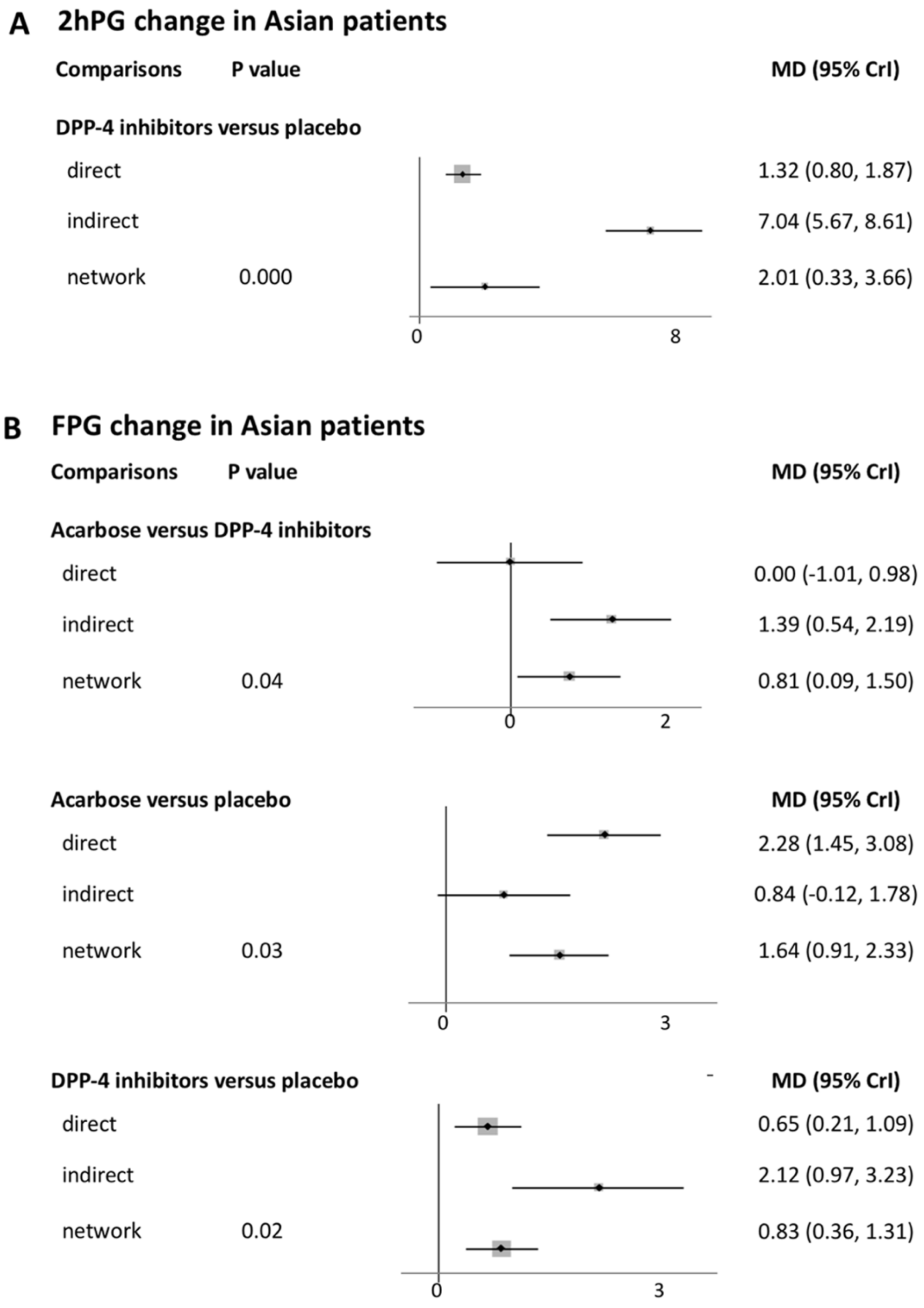

Figure S9. Inconsistent results of network meta-analysis in comparisons between direct and indirect evidences in (**A**) 2-hour postprandial glucose change in Asian patients, and (**B**) fasting plasma glucose change in Asian patients. DPP-4, dipeptidyl peptidase-4; 2hPG, 2-hour postprandial glucose; FPG, fasting plasma glucose; MD, mean difference; CrI, credible interval.
