# Supplementary material for: Acarbose With Comparable Glucose-Lowering but Superior Weight-Loss Efficacy to Dipeptidyl Peptidase-4 Inhibitors: A Systematic Review and Network Meta-Analysis of Randomized Controlled Trials
Source: Front Endocrinol (Lausanne). 2020 Jun 5;11:288. doi: 10.3389/fendo.2020.00288 (PMC7291873; doi:10.3389/fendo.2020.00288)
Supplement: Supplementary file 3 [file Table_2.PDF]

Table S2. Characteristics of the 75 included studies in systematic review.

	Study	Setting and design	Ethnicity	Treatment	Sample size	Treatment duration	Diabetes duration (years (SD/95%CI))	mean age (years (SD/95%CI))	Female (%)	HbA1c (% (SD/95%CI)) at baseline	BMI (kg.m-2 (SD/95%CI)) at baseline	Outcomes
**Acarbose versus DPP-4 inhibitors in pair-wise studies (n = 13)**												
1	Pan, et al 2008	Design: double-blind, randomized, active-controlled, parallel-group study for drug-naive T2D patients	Asian: 91.8%	acarbose 100 mg tid	220	24 weeks	1.3 (2.4)	51.9 (10.3)	36.8	8.6 (1.0)	25.8 (3.5)	changes from baseline in HbA1c, FPG, body weight; achievements of HbA1c < 7% and < 6.5% targets; safety events
		Locations: China, Romania and Spain	Asian: 90.5%	vildagliptin 50 mg bid	440	24 weeks	1.2 (2.4)	51.8 (10.1)	39.9	8.6 (0.9)	26.4 (3.6)	
2	Wang, et al 2015	Design: randomized, active-controlled study for aged T2D patients inadequately controlled with metformin alone	Chinese	acarbose 50 mg tid + metformin	40	1 year	14.2 (4.2)	65.1 (3.1)	52.5	8.3 (0.37)	—	changes from baseline in HbA1c, FPG, PPG, body weight, lipids profile; achievements of HbA1c < 7.5% and < 7% targets
		Location: China	Chinese	saxagliptin 5 mg qd + metformin	41	1 year	12.3 (4.5)	64.3 (2.6)	56.1	8.26 (0.49)	—	
3	Du, et al 2017	Design: multicentre, randomized, parallel-group, open-label Phase IV study in patients with T2D inadequately controlled with metformin monotherapy	Chinese	acarbose 100 mg tid + metformin	243	24 weeks	5.3 (4.76)	56.5 (10.81)	43.2	8.16 (0.81)	26.3 (3.49)	changes from baseline in HbA1c, FPG, PPG, body weight; achievement of HbA1c < 7% target
		Location: China	Chinese	saxagliptin 5 mg qd + metformin	238	24 weeks	5.1 (4.40)	54.7 (10.51)	38.2	8.23 (0.85)	26.4 (3.47)	
4	Bao, et al 2014	Design: randomized, active-controlled study for drug-naive T2D patients	Chinese	acarbose 50 mg tid	64	24 weeks	—	48.2 (6.1)	46.9	7.6 (0.8)	24.4 (3.2)	changes from baseline in HbA1c, FPG, PPG, BMI, ghrelin, lipids profile; safety events
		Location: China	Chinese	sitagliptin 100 mg qd	64	24 weeks	—	50.3 (5.4)	50.0	7.8 (0.8)	24.9 (3.7)	
5	Guo, et al 2015	Design: randomized, active-controlled study for T2D patients with early diabetic nephropathy	Chinese	acarbose 200 mg/d + Novolin 30R	53	48 weeks	4.96 (3.28)	57.48 (8.56)	50.94	7.88 (1.58)	27.64 (2.95)	changes from baseline in HbA1c, FPG, blood pressures
		Location: China	Chinese	sitagliptin 100 mg/d + Novolin 30R	52	48 weeks	5.15 (2.75)	56.45 (7.56)	51.92	7.98 (1.64)	26.58 (3.25)	
6	Li, et al 2016	Design: randomized, active-controlled study for T2D patients	Chinese	acarbose 100 mg tid	50	6 months	2.6 (0.7)	51.8 (10.2)	42	7.8 (0.5)	26.98 (2.97)	changes from baseline in HbA1c, FPG, liver function, renal function; safety events
		Location: China	Chinese	sitagliptin 100 mg qd	50	6 months	2.8 (0.8)	50.2 (8.8)	38	7.9 (0.6)	27.18 (2.29)	
7	Xue, et al 2016	Design: randomized, active-controlled study for T2D patients	Chinese	acarbose 50 mg tid + metformin 0.5 g tid + insulin	35	24 weeks	3.7 (1.4)	60.1 (4.2)	48.57	8.12 (1.46)	28.1 (1.4)	changes from baseline in HbA1c, FPG, PPG, BMI, lipids profile,

		Location: China	Chinese	sitagliptin 100 mg qd + metformin 0.5 g tid + insulin	35	24 weeks	3.19 (1.12)	59.6 (4.3)	57.14	7.96 (1.39)	27.5 (1.2)	insulin dosages; safety events
8	Zhang, et al 2016	Design: randomized, active-controlled study for T2D patients inadequately controlled with metformin monotherapy	Chinese	acarbose 50 mg tid + metformin 0.5 g tid	30	24 weeks	newly diagnosed T2D	58.3 (7.8)	50.0	8.3 (1.2)	28.1 (0.7)	changes from baseline in HbA1c, FPG, BMI, blood pressures; safety events
		Location: China	Chinese	sitagliptin 100 mg qd + metformin 0.5 g tid	30	24 weeks		58.0 (8.7)	46.67	8.4 (1.1)	27.9 (0.6)	
9	Gao, et al 2015	Design: randomized, active-controlled study for aged T2D patients inadequately controlled with insulin glargine monotherapy	Chinese	acarbose 50 mg tid + insulin glargine	30	24 weeks	7.19 (4.21)	70.45 (2.55)	43.33	9.85 (2.34)	25.87 (1.72)	changes from baseline in HbA1c, FPG, PPG, insulin dosages, body weight, blood pressures, lipids profile; safety events
		Location: China	Chinese	saxagliptin 5 mg qd + insulin glargine	30	24 weeks	7.65 (3.13)	69.32 (3.85)	50.0	9.32 (1.55)	26.32 (1.68)	
10	Su, et al 2016	Design: randomized, active-controlled study for T2D patients inadequately controlled with insulin glargine monotherapy	Chinese	acarbose 50 mg tid + insulin glargine	51	24 weeks	6.5 (2.9)	64.8 (4.2)	39.22	9.38 (1.59)	26.4 (2.8)	changes from baseline in HbA1c, FPG, insulin dosages; safety events
		Location: China	Chinese	saxagliptin 5 mg qd + insulin glargine	51	24 weeks	6.7 (2.4)	65.1 (4.9)	43.14	9.41 (1.65)	27.1 (2.2)	
11	Li, et al 2016	Design: randomized, active-controlled study for T2D patients inadequately controlled with metformin monotherapy	Chinese	acarbose + metformin	50	24 weeks	less than 5 years	52.4 (3.8)	50.0	7.8 (—)	23.9 (1.0)	changes from baseline in FPG, PPG
		Location: China	Chinese	alogliptin + metformin	50	24 weeks		53.1 (3.0)	50.0	7.9 (—)	24.3 (1.5)	
12	Zhang, et al 2016	Design: randomized, active-controlled study for T2D patients inadequately controlled with metformin and pioglitazone	Chinese	acarbose 50 mg tid + metformin 0.5 g tid + pioglitazone 30 mg qd	25	6 months	—	68.2 (6.3)	44.0	10.4 (1.4)	25.4 (2.5)	changes from baseline in HbA1c, FPG, PPG, BMI; safety events
		Location: China	Chinese	alogliptin 25 mg qd + metformin 0.5 g tid + pioglitazone 30 mg qd	25	6 months	—	67.8 (5.2)	40.0	10.6 (1.6)	25.1 (2.3)	
13	Duan, et al 2016	Design: randomized, active-controlled study for T2D patients	NA	acarbose 50 mg tid + metformin 0.5 g tid	103	1 year	1.0 (0.1)	48.88 (2.91)	46.6	8.12 (1.09)	27.32 (1.81)	changes from baseline in HbA1c, FPG, PPG, BMI, lipids profile; safety events
		Location: China	NA	sitagliptin 100 mg qd + metformin 0.5 g tid	105	1 year	1.0 (0.09)	50.32 (3.21)	43.8	7.94 (0.96)	27.10 (1.73)	

**Acarbose versus placebo (n = 15)**											
14 Hanefeld, et al 1991	Design: randomized double-blind placebo-controlled study for T2D patients with diet treatment alone	NA	acarbose 100 mg tid	47	24 weeks	5.83 (range: minimum—maximum 0.67—19.58)	60 (43—70)	51	9.3 (1.38)	27.4 (20.6—36.1)	changes from baseline in HbA1c, FPG, PPG, insulin and c-peptide levels, blood pressure, lipids profile; safety events
	Location: Germany	NA	placebo	47	24 weeks	4.08 (range: minimum—maximum 0.42—16.42)	59 (43—70)	47	9.40 (1.14)	27.7 (19.4—37.0)	
15 Coniff, et al 1995	Design: multicenter, randomized double-blind study with four parallel treatments for T2D patients	non-Asian	acarbose 200 mg tid	67	24 weeks	5.1 (—)	56.2 (—)	61	6.88 (—)	29.7 (—)	changes from baseline in HbA1c, FPG, PPG, lipids profile; safety events
		non-Asian	placebo	62	24 weeks	5.5 (—)	56.3 (—)	48	7.10 (—)	29.9 (—)	
	Location: U.S.	non-Asian	tolbutamide 250—1000 mg/d	66	24 weeks	5.6 (—)	55.4 (—)	44	6.95 (—)	29.5 (—)	
		non-Asian	acarbose + tolbutamide	60	24 weeks	5.3 (—)	55.7 (—)	48	6.73 (—)	30.4 (—)	
16 Hoffmann, et al 1994	Design: randomized double-blind study with three parallel treatments for T2D patients with diet treatment alone	NA	acarbose 100 mg tid	28	24 weeks	1.06 (0.9)	58.8 (6.9)	54	8.29 (0.42)	26.5 (1.6)	changes from baseline in HbA1c, insulin level, lipids profile; safety events
		NA	placebo	30	24 weeks	1.01 (0.9)	56.9 (6.7)	60	8.29 (0.37)	26.8 (1.5)	
	Location: Germany	NA	glibenclamide 4.3 mg/d	27	24 weeks	1.47 (1.09)	59.5 (5.7)	52	8.3 (0.37)	26.5 (2.1)	
17 Hoffmann, et al 1997	Design: randomized double-blind study with three parallel treatments for T2D patients	NA	acarbose 100 mg tid	31	24 weeks	3.08 (2.27)	58.9 (9.4)	81	9.6 (0.9)	26.4 (2.7)	changes from baseline in HbA1c, body weight, lipids profile; safety events
		NA	placebo	32	24 weeks	3.6 (2.83)	60.2 (8.6)	62	9.4 (0.9)	26.3 (2.2)	
	Location: Germany	NA	metformin 0.85 g bid	31	24 weeks	2.08 (1.45)	55.9 (7.8)	55	9.7 (0.9)	27.4 (2.2)	
18 Chan, et al 1998	Design: multicenter randomized, double-blind placebo-controlled study for T2D patients with dietary failure	Asian	acarbose 100 mg tid	63	24 weeks	2.7 (3.5)	52.8 (10.2)	49.21	8.2 (1.0)	25.4 (3.9)	changes from baseline in HbA1c, FPG, PPG, body weight, BMI, blood pressures, lipids profile; safety events
	Locations: Taiwan, Hong Kong, Philippines, Korea, Singapore, Malaysia	Asian	placebo	63	24 weeks	2.1 (3.4)	54.0 (10.0)	49.21	8.6 (1.1)	25.6 (3.8)	
19 Holman, et al 1999	Design: multicenter randomized, double-blind placebo-controlled study for T2D patients with diet and/or preexisting therapies	NA	acarbose 100 mg tid	973	3 years	7.9 (2.9)	60 (9)	27	8.7 (6.8—11.2_	29.8 (5.6)	changes from baseline in HbA1c; safety events
	Location: England	NA	placebo	973	3 years	8.0 (2.8)	60 (9)		8.7 (6.8—11.0_	29.6 (5.7)	
20 Josse, et al 2003	Design: randomized, double-blind placebo-controlled study for aged T2D patients with diet treatment alone	non-Asian: 96%	acarbose 50—100 mg tid	93	12 months	5.8 (6.75)	69.7 (4.82)	30.11	7.4 (0.96)	28.3 (3.86)	changes from baseline in HbA1c, FPG, insulin level; safety events

		Location: Canada	non-Asian: 95%	placebo	99	12 months	4.8 (4.97)	70.3 (4.97)	39.39	7.3 (0.99)	28.6 (3.98)	
21	Kirkman, et al 2006	Design: randomized, double-blind placebo-controlled study for patients with early T2D	non-Asian: 98.2%	acarbose 100 mg tid	109	1 year	—	53.7 (11)	67	6.35 (0.65)	35.1 (7.2)	changes from baseline in HbA1c, PPG, insulin level
		Location: U.S.	non-Asian: 99.1%	placebo	110	1 year	—	53.7 (11.7)	65.4	6.33 (0.63)	35.2 (7.1)	
22	Wolever, et al 1997	Design: multicenter, randomized, double-blind placebo-controlled study for T2D patients with diet, or metformin, or sulfonylueas, or insulin treatments	non-Asian: 94.8%	acarbose 200 mg tid	30	1 year	5.2 (5.26)	57.2 (9.65)	37.66	6.97 (1.6)	28.8 (4.39)	changes from baseline in HbA1c, FPG, body weight, lipids profile; achievement of HbA1c < 7% target; safety events
				placebo	37	1 year				6.72 (1.08)		
			non-Asian: 92.8%	acarbose 200 mg tid + metformin	35	1 year	8.8 (5.47)	57.4 (10.02)	36.1	8.19 (1.7)	29.4 (5.47)	
				placebo + metformin	39	1 year				7.76 (1.48)		
			non-Asian: 94.8%	acarbose 200 mg tid + sulfonylureas	49	1 year	9.4 (7.1)	58.4 (9.13)	43.7	8.15 (1.44)	27.8 (4.06)	
				placebo + sulfonylureas	47	1 year				8.02 (1.37)		
		Location: Canada	non-Asian: 92.3%	acarbose 200 mg tid + insulin	35	1 year	12.9 (7.63)	56.6 (8.59)	42.9	7.69 (0.9)	30.2 (4.77)	
				placebo + insulin	44	1 year				7.84 (1.17)		
23	Zheng, et al 1995	Design: randomized, double-blind placebo-controlled study for T2D patients with diet therapy	Chinese	acarbose 100 mg tid	39	24 weeks	4.1 (2.8)	49.6 (6.9)	48.72	9.85 (2.37)	24.62 (2.69)	changes from baseline in HbA1c, FPG, BMI, insulin level; safety events
		Location: China	Chinese	placebo	38	24 weeks	4.2 (3.6)	49.0 (6.6)	47.37	9.82 (2.16)	26.19 (3.27)	
24	Fischer, et al 1998	Design: multicentre, randomised, double-blind, placebo-controlled, five-arm study for drug-naive T2D patients	NA	acarbose 25 mg tid	86	24 weeks	2.17 (median)	58.5 (8.4)	47	7.42 (1.09)	27.3 (3.5)	changes from baseline in HbA1c; safety events
			NA	acarbose 50 mg tid	88	24 weeks	1.67 (median)	55.5 (9.6)	51	7.52 (1.09)	27.6 (3.5)	
			NA	acarbose 100 mg tid	78	24 weeks	1.42 (median)	56.8 (9.4)	41	7.43 (1.1)	27.6 (3.7)	
		Locations: Austria, Croatia, Germany, Hungary, Italy	NA	acarbose 200 mg tid	87	24 weeks	1.75 (median)	59.4 (8.6)	49	7.51 (1.1)	27.2 (3.3)	
			NA	placebo	81	24 weeks	2 (median)	52.7 (8.7)	47	7.26 (1.09)	26.9 (2.9)	
25	Wu, et al 2003	Design: randomized, double-blind placebo-controlled study for T2D patients with diet therapy	NA	acarbose 100 mg tid	80	24 weeks	4.2 (2.1)	50 (7)	43.75	9.8 (2.1)	26.5 (2.3)	changes from baseline in HbA1c, FPG, PPG, BMI, insulin level
		Location: China	NA	placebo	80	24 weeks	4.3 (2.4)	50 (6)	41.25	9.6 (2.7)	27.2 (2.5)	
26	Hasche, et al 1999	Design: randomized, double-blind placebo-controlled study for T2D patients with diet therapy	NA	acarbose 100 mg tid	36	104 weeks	0.96 (—)	63.8 (9.8)	52.8	8.5 (0.7)	26.1 (2.9)	changes from baseline in HbA1c, FPG, PPG, body weight, lipids profile; safety events
		Location: Germany	NA	placebo	38	104 weeks	1.0 (—)	63.1 (10.5)	50.0	8.3 (0.7)	26.7 (2.8)	

27	Braun, et al 1996	Design: randomized, double-blind placebo-controlled study for T2D patients with diet therapy	NA	acarbose 100 mg tid	42	24 weeks	1.33 (range: minimum—maximum 0.25—4)	60 (range: minimum—maximum 47 — 75)	38.1	10.0 (1.5)	26 range: minimum—maximum 20 — 32)	changes from baseline in HbA1c, FPG, body weight, lipids profile; safety events
		Location: Germany	NA	placebo	44	24 weeks	1.42 (range: minimum—maximum 0.25— 5)	61 (range: minimum—maximum 42 — 74)	45.5	9.9 (1.5)	26 range: minimum—maximum 22 — 31)	
28	Kovacevic, et al 1997	Design: multicentric, randomized, double-blind, controlled study for T2D patients with diet therapy	NA	acarbose 100 mg tid	33	24 weeks	4.5 (—)	57.54 (8.08)	54.0	8.3 (0.7)	28.73 (2.83)	changes from baseline in HbA1c, FPG, insulin level, lipids profile; safety events
			NA	glibenclamide 3.5—10.5 mg/d	33	24 weeks				9.0 (1.0)		
		Location: Croatia	NA	placebo	31	24 weeks				8.3 (1.09)		
**Acarbose versus sulfonylureas (n = 1)**												
29	Hu, et al 2012	Design: randomized, non-inferiority controlled study for newly diagnosed T2D patients	Chinese	acarbose 50 —100 mg tid	34	24 weeks	newly diagnosed T2D	49.34 (6.07)	55.88	7.45 (0.72)	26.98 (1.72)	changes from baseline in HbA1c, FPG, PPG, BMI, lipids profile; safety events
		Location: China	Chinese	glimepiride 1 —6 mg/d	34	24 weeks		49.44 (6.32)	55.88	7.64 (0.81)	27.06 (2.33)	
**Acarbose versus metformin (n = 4)**												
30	Yang, et al 2013	Design: randomized, open-label, non-inferiority trial for newly diagnosed T2D patients	Chinese	acarbose 100 mg tid	361	48 weeks	0.22 (0.22)	50.6 (9.2)	39	7.49 (1.26)	25.5 (2.7)	changes from baseline in HbA1c, FPG, PPG, body weight, lipids profile; achievements of HbA1c < 7% and < 6.5% targets; safety events
		Location: China	Chinese	metformin 0.5 g tid	351	48 weeks	0.26 (0.25)	50.2 (9.3)	40	7.59 (1.22)	25.7 (2.6)	
31	Wang, et al 2011	Design: randomized, non-inferiority trial for newly diagnosed T2D patients	Chinese	acarbose 300 mg/d	34	48 weeks	newly diagnosed T2D	50.34 (7.07)	55.9	7.45 (0.72)	26.98 (1.72)	changes from baseline in HbA1c, FPG, PPG, BMI, lipids profile; safety events
		Location: China	Chinese	metformin 1.5 g/d	34	48 weeks		50.44 (7.33)	55.9	7.64 (0.81)	27.06 (2.33)	
32	Rong, et al 2008	Design: randomized, non-inferiority trial for drug-naive T2D patients	Chinese	acarbose 100 mg tid	28	24 weeks	3.6 (0.7)	56.5 (12.0)	38.3	—	—	changes from baseline in FPG, PPG, insulin levels; safety events
		Location: China	Chinese	metformin 0.5 g tid	32	24 weeks				—	—	
33	Zhu, et al 2011	Design: randomized, non-inferiority trial for newly diagnosed T2D patients	Chinese	acarbose 50 mg tid	32	6 months	newly diagnosed T2D	52 (10)	42.7	8.3 (1.3)	—	changes from baseline in HbA1c, FPG, PPG, lipids profile; safety events
		Location: China	Chinese	metformin 0.5 g tid	33	6 months				8.2 (1.1)	—	
**Acarbose versus thiazolidinediones (n = 1)**												
34	Göke, et al 2002	Design: open-label, randomized, parallel-group multi-center study for T2D patients	NA	acarbose 100 mg tid	136	26 weeks	3.28 (2.79)	58.8 (9.1)	45.6	9.03 (1.32)	30.8 (4.4)	changes from baseline in HbA1c, FPG, lipids profile; achievements of HbA1c < 7% and <
		Location: Germany	NA	pioglitazone 15 mg tid	129	26 weeks	3.17 (3.08)	58.9 (9.1)	46.5	8.98 (1.20)	30.9 (5.3)	

												6.5% targets; safety events
**Acarbose as add-on drug (n = 1)**												
35	A, et al 2015	Design: randomized, open-label, controlled study for T2D patients	Asian: 100%	acarbose 50 mg tid + metformin 0.5 g bid	41	6 months	10.08 (1.87)	57.26 (10.37)	43.9	8.96 (1.36)	21.39 (4.37)	changes from baseline in HbA1c, FPG, PPG, lipids profile; safety events
		Location: China	Asian: 100%	metformin 0.5 g bid	41	6 months	10.03 (1.65)	57.95 (11.16)	41.5	8.92 (1.35)	21.18 (4.16)	
**DPP-4 inhibitors versus placebo (n = 26)**												
36	Defronzo, et al 2008	Design: double-blind, randomized, placebo-controlled, study for drug-naive T2D patients	White: 66.9%	alogliptin 12.5 mg qd	133	26 weeks	—	53.4 (11.1)	51.1	—	—	changes from baseline in HbA1c, FPG, body weight, lipids profile; achievements of HbA1c < 7% and < 6.5% targets; safety events
				alogliptin 25 mg qd	132	26 weeks			41.2			
		Locations: 16 countries		placebo	65	26 weeks			58.2			
37	Inagaki, et al 2015	Design: double-blind, randomized, active-controlled, study for T2D patients with lifestyle intervention	NA	alogliptin 25 mg qd	92	24 weeks	7.07 (5.94)	60 (IQR: 53-65)	25	7.87 (0.86)	24.7 (3.79)	changes from baseline in HbA1c, FPG, PPG; safety events
			NA	trelagliptin 100 mg qw	101	24 weeks	4.17 (5.94)	58 (IQR: 52-65)	28	7.73 (0.85)	25.4 (4.42)	
		Location: Japan	NA	placebo	50	24 weeks	7.55 (5.50)	62 (IQR: 54-67)	14	7.72 (0.77)	24.6 (4.27)	
38	Ji, et al 2017	Design: double-blind, randomized, placebo-controlled, multicentre study for T2D patients with lifestyle intervention	Asian: 99.4%	alogliptin 12.5 mg bid	163	26 weeks	—	55.4 (9.62)	39.9	8.48 (0.71)	26.16 (3.92)	changes from baseline in HbA1c; achievements of HbA1c < 7% and < 6.5% targets; safety events
			Asian: 98.8%	placebo	163	26 weeks		52.2 (10.17)	41.7	8.21(0.77)	26.56 (4.22)	
		Locations: China, Malaysia, South Korea	Asian: 99.4%	metformin 0.5 g bid	162	26 weeks		53.6 (9.91)	49.4	8.40 (0.78)	26.30 (3.57)	
			Asian: 100%	alogliptin 12.5 mg bid + metformin 0.5 g bid	159	26 weeks		53.4 (10.46)	42.8	8.39 (0.81)	26.16 (3.51)	
39	Yang, et al 2015	Design: multicentre, double-blind, randomized, placebo-controlled study for drug-naive T2D patients	NA	anagliptin 100 mg bid	37	24 weeks	3.17 (5.53)	54.43 (9.86)	59.46	7.13 (0.72)	24.60 (3.01)	changes from baseline in HbA1c, FPG, body weight, BMI, lipids profile; safety events
			NA	anagliptin 200 mg bid	33	24 weeks	3.43 (3.40)	57.70 (9.71)	40	7.19 (0.73)	24.97 (2.64)	
		Location: Republic of Korea (South Korea)	NA	placebo	39	24 weeks	4.14 (4.10)	56.74 (9.72)	36.84	7.11 (0.63)	25.44 (3.19)	
40	Park, et al 2017	Design: multicentre, double-blind, randomized, placebo-controlled study for T2D patients	NA	evogliptin 5 mg qd	80	24 weeks	4.74 (3.81)	57.6 (11.0)	51.2	7.21 (0.56)	25.6 (3.2)	changes from baseline in body weight, lipids profile; achievement of HbA1c < 6.5% target; safety events
		Location: Republic of Korea	NA	placebo	80	24 weeks	4.25 (4.10)	56.8 (9.8)	42.5	7.20 (0.63)	25.4 (3.4)	
41	Yang, et al 2012	Design: multicentre, double-blind, randomized, placebo-controlled study for T2D patients	NA	gemigliptin 50 mg qd	87	24 weeks	3.24 (3.84)	54 (IQR: 49—60_	43.7	8.2 (1.0)	25.4 (IQR: 22.8—28.1)	achievements of HbA1c < 7% and < 6.5% targets; safety events
		Locations: Republic of Korea, India	NA	placebo	87	24 weeks	2.86 (4.36)	52 (IQR: 45—60_	40.2	8.3 (1.1)	26.7 (IQR: 23.6—29.1)	
42	Del Prato, et al 2011	Design: multicentre, double-blind, randomized, placebo-	non-Asian: 53.6%	linagliptin 5 mg qd	336	24 weeks	—	56.4 (10.1)	51.2	8.0 (0.91)	29.04 (4.80)	changes from baseline in HbA1c, FPG,

		controlled study for T2D patients										PPG; safety events
		Locations: 11 countries	non-Asian: 54.5%	placebo	167	24 weeks		54.4 (10.3)	52.7	8.0 (0.87)	29.08 (4.84)	
43	Chen, et al 2015	Design: multicentre, double-blind, randomized, placebo-controlled study for T2D patients	Asian: 100%	linagliptin 5 mg qd	200	24 weeks	—	54.6 (10.1)	42	7.95 (0.89)	25.5 (3.3)	changes from baseline in HbA1c; achievements of HbA1c < 7% and < 6.5% targets; safety events
		Locations: China, Malaysia, Philippines	Asian: 100%	placebo	99	24 weeks		54.1 (9.3)	40.4	8.09 (0.91)	25.1 (3.4)	
44	Wu, et al 2015	Design: double-blind, randomized, placebo-controlled study for drug-naïve T2D patients	Chinese	linagliptin 5 mg qd	34	24 weeks	newly-diagnosed T2D	52.5 (11.0)	34.3	7.97 (0.68)	24.37 (2.09)	changes from baseline in HbA1c, FPG, PPG, body weight, lipids profile; safety events
		Location: China	Chinese	placebo	23	24 weeks		51.2 (7.5)	50	8.00 (0.69)	24.11 (2.28)	
45	Rosenstock, et al 2009	Design: double-blind, randomized, placebo-controlled study for drug-naïve T2D patients	non-Asian: 95.1%	saxagliptin 2.5 mg qd	102	24 weeks	3.1 (3.5)	53.27 (10.06)	43.1	7.9 (0.9)	31.90 (4.82)	changes from baseline in HbA1c, FPG; achievement of HbA1c < 7% target; safety events
			non-Asian: 96.2%	saxagliptin 5 mg qd	106	24 weeks	2.5 (3.3)	53.91 (11.57)	49.1	8.0 (1.1)	32.24 (4.50)	
			non-Asian: 93.9%	saxagliptin 10 mg qd	98	24 weeks	2.3 (3.1)	52.72 (11.27)	54.1	7.8 (0.9)	31.71 (4.71)	
		Locations: US, Mexico	non-Asian: 96.8%	placebo	95	24 weeks	2.3 (2.7)	53.91 (12.32)	50.5	7.9 (0.9)	30.93 (4.26)	
46	Frederich, et al 2012	Design: double-blind, randomized, placebo-controlled study for drug-naïve T2D patients	non-Asian: 75.7%	saxagliptin 2.5 mg qd	74	76 weeks	1.2 (1.6)	55.2 (10.44)	66.2	8.0 (0.8)	30.4 (4.84)	changes from baseline in HbA1c, FPG; achievement of HbA1c < 7% target; safety events
			non-Asian: 73%	saxagliptin 5 mg qd (AM)	74	76 weeks	1.7 (2.4)	54.7 (9.71)	48.6	8.0 (0.9)	31.0 (5.23)	
			non-Asian: 80.3%	saxagliptin 2.5/5 mg qd	71	76 weeks	2.0 (2.9)	54.3 (10.93)	47.9	8.0 (1.1)	30.6 (4.72)	
		Locations: US, Russia, India, Taiwan	non-Asian: 77.8%	saxagliptin 5 mg qd (PM)	72	76 weeks	2.0 (5.2)	55.1 (10.35)	54.2	7.9 (0.9)	29.6 (5.37)	
			non-Asian: 77%	placebo	74	76 weeks	1.7 (2.8)	55.6 (10.32)	52.7	7.8 (1.0)	31.1 (4.54)	
47	Pan, et al 2012	Design: multicentre, double-blind, randomized, placebo-controlled study for drug-naïve T2D patients	Asian: 100%	saxagliptin 5 mg qd	284	24 weeks	0.8 (1.4)	51.2 (10.0)	34.7	8.1 (0.8)	25.9 (3.4)	changes from baseline in HbA1c, FPG, 2hPG; achievement of HbA1c < 7% target; safety events
		Locations: China, India, Philippines, South Korea	Asian: 100%	placebo	284	24 weeks	1.2 (2.6)	51.6 (10.3)	45.4	8.2 (0.8)	25.9 (3.7)	
48	Kumar, et al 2014	Design: multicentre, double-blind, randomized, placebo-controlled study for drug-naïve T2D patients	Asian: 100%	saxagliptin 5 mg qd	107	24 weeks	0.8 (1.2)	49.1 (8.8)	46.7	8.3 (0.8)	26.6 (4.2)	changes from baseline in HbA1c, FPG; achievements of HbA1c <

		Location: India	Asian: 100%	placebo	106	24 weeks	1.0 (1.4)	48.3 (9.6)	40.6	8.3 (0.7)	27.0 (4.3)	7% and < 6.5% targets; safety events
49	Aschner, et al 2006	Design: multicentre, double-blind, randomized, placebo-controlled study for T2D patients	non-Asian: 86.6%	sitagliptin 100 mg qd	238	24 weeks	4.3 (4.9)	53.4 (9.5)	42.9	8.0 (0.9)	30.3 (5.2)	changes from baseline in HbA1c, FPG, PPG; achievement of HbA1c < 7% target; safety events
			non-Asian: 85.2%	sitagliptin 200 mg qd	250	24 weeks	4.3 (4.7)	54.9 (10.1)	53.2	8.1 (0.9)	30.3 (5.4)	
		Locations: 18 countries	non-Asian: 86.6%	placebo	253	24 weeks	4.6 (4.7)	54.3 (10.1)	48.6	8.0 (0.8)	30.8 (5.5)	
50	Goldstein, et al 2007	Design: multicentre, double-blind, randomized, placebo-controlled, parallel-group study for T2D patients	non-Asian: 96.6%	sitagliptin 100 mg qd	179	24 weeks	4.4 (4.6)	53.3 (10.2)	48.0	8.9 (1.0)	31.2 (5.9)	changes from baseline in HbA1c, FPG, PPG; achievements of HbA1c < 7% and < 6.5% targets; safety events
			non-Asian: 93.2%	placebo	176	24 weeks	4.6 (4.9)	53.6 (10.0)	47.2	8.7 (1.0)	32.5 (6.7)	
			non-Asian: 92.3%	metformin 0.5 g bid	182	24 weeks	4.5 (3.9)	53.4 (10.2)	51.1	8.9 (1.0)	32.1 (6.8)	
			non-Asian: 94.5%	metformin 1 g bid	182	24 weeks	4.4 (4.4)	53.2 (9.6)	54.9	8.7 (0.9)	32.2 (7.1)	
			non-Asian: 95.3%	sitagliptin 50 mg bid + metformin 0.5 g bid	190	24 weeks	4.5 (4.7)	54.1 (10.0)	44.7	8.8 (1.0)	32.1 (6.7)	
		Locations: 15 countries	non-Asian: 94.0%	sitagliptin 50 mg bid + metformin 1 g bid	182	24 weeks	4.4 (4.2)	53.3 (9.6)	57.7	8.7 (0.9)	32.4 (6.6)	
51	Barzilai, et al 2011	Design: multicentre, double-blind, randomized, placebo-controlled study for aged T2D patients	non-Asian: 97.1%	sitagliptin 50/100 mg qd	102	24 weeks	7.2 (7.3)	71.6 (6.1)	52.9	7.8 (0.8)	30.8 (5.9)	changes from baseline in HbA1c, FPG, PPG; achievement of HbA1c < 7% target; safety events
		Location: U.S.	non-Asian: 97.1%	placebo	104	24 weeks	7.0 (7.5)	72.1 (6.0)	52.9	7.8 (0.7)	31.1 (7.2)	
52	Ji, et al 2016	Design: multicentre, double-blind, randomized, placebo-controlled study for T2D patients	Asian: 100%	sitagliptin 100 mg qd	120	24 weeks	1.1 (0.2)	51.7 (10.2)	38.3	8.7 (1.1)	26.0 (3.5)	changes from baseline in HbA1c, FPG, PPG, body weight; achievements of HbA1c < 7% and < 6.5% targets; safety events
			Asian: 100%	placebo	127	24 weeks	1.1 (0.2)	53.6 (9.7)	31.5	9.0 (1.1)	25.4 (3.4)	
			Asian: 100%	metformin 0.5 g bid	126	24 weeks	1.0 (0.2)	52.6 (9.5)	45.2	8.7 (1.0)	26.0 (3.7)	
			Asian: 100%	metformin 0.85 g bid	124	24 weeks	1.1 (0.2)	53.0 (10.3)	39.5	8.7 (1.1)	25.8 (3.5)	
			Asian: 100%	sitagliptin 100 mg qd + metformin 0.5 g bid	122	24 weeks	1.1 (0.3)	52.6 (11.3)	30.3	8.5 (1.0)	26.1 (3.4)	
		Location: China	Asian: 100%	sitagliptin 100 mg qd + metformin 0.85 g bid	125	24 weeks	1.1 (0.3)	52.4 (9.3)	46.4	8.6 (0.9)	25.4 (3.1)	

53	Gantz, et al 2017	Design: multicentre, double-blind, randomized, placebo- and sitagliptin-controlled study for T2D patients	Asian: 100%	omarigliptin 25 mg qw	166	24 weeks	7.4 (5.5)	60 (11)	37.3	7.9 (0.7)	25.2 (3.7)	changes from baseline in HbA1c, FPG, PPG, body weight; achievements of HbA1c < 7% and < 6.5% targets; safety events
			Asian: 100%	sitagliptin 50 mg qd	165	24 weeks	7.4 (5.3)	60 (9)	30.3	8.0 (0.8)	25.4 (4.2)	
		Location: Japan	Asian: 100%	placebo	83	24 weeks	8.6 (5.1)	61 (9)	31.3	8.1 (0.7)	24.3 (3.3)	
54	Gupta, et al 2017	Design: multicentre, double-blind, randomized, placebo-controlled study for drug-naïve T2D patients	Asian: 100%	sitagliptin 100 mg qd	27	76 weeks	1—10 years	49.5 (9.4)	44.4	8.31 (0.68)	26.47 (3.61)	changes from baseline in HbA1c, FPG, body weight; achievement of HbA1c < 7% target; safety events
		Location: India	Asian: 100%	placebo	28	76 weeks		48.7 (8.7)	57.1	7.92 (0.70)	26.85 (4.49)	
55	Zhao, et al 2017	Design: double-blind, randomized, placebo-controlled study for T2D patients	Asian: 100%	sitagliptin 100 mg qd	50	42 weeks	5.7 (4.2)	68.5 (8.0)	36	7.6 (0.7)	28.9 (2.4)	changes from baseline in HbA1c, BMI, body weight; safety events
		Location: China	Asian: 100%	placebo	50	42 weeks	5.8 (4.6)	69.1 (8.4)	42	7.6 (0.9)	28.2 (2.9)	
56	Hong, et al 2016	Design: double-blind, randomized, placebo-controlled study for T2D patients	Asian: 100%	teneligliptin 20 mg qd	99	24 weeks	4.59 (3.87)	56.64 (10.07)	47.47	7.63 (0.69)	24.96 (2.51)	changes from baseline in HbA1c, FPG, body weight; achievements of HbA1c < 7% and < 6.5% targets; safety events
		Location: Korea	Asian: 100%	placebo	43	24 weeks	4.59 (3.94)	57.93 (11.90)	34.88	7.77 (0.81)	25.07 (3.23)	
57	Dejager, et al 2007	Design: double-blind, randomized, placebo-controlled study for drug-naive T2D patients	non-Asian: 96.2%	vildagliptin 50 mg qd	104	24 weeks	2.1 (3.6)	55.3 (11.4)	58.7	8.2 (0.8)	32.9 (6.0)	changes from baseline in HbA1c, body weight; safety events
			non-Asian: 66.6%	vildagliptin 50 mg bid	90	24 weeks	2.1 (3.3)	52.8 (9.6)	53.3	8.6 (0.8)	33.3 (4.8)	
		Locations: U.S., Russia, Tunisia	non-Asian: 95.6%	vildagliptin 100 mg qd	92	24 weeks	2.4 (4.2)	53.6 (10.8)	46.7	8.4 (0.8)	32.4 (6.1)	
			non-Asian: 93.6%	placebo	94	24 weeks	1.6 (2.5)	52.2 (11.2)	52.1	8.4 (0.8)	32.6 (5.6)	
58	Pi-Sunyer, et al 2007	Design: double-blind, randomized, placebo-controlled study for drug-naive T2D patients	non-Asian: 80.7%	vildagliptin 50 mg qd	88	24 weeks	1.8 (2.7)	50.6 (10.4)	44.3	8.4 (0.9)	31.9 (5.4)	changes from baseline in HbA1c, FPG, body weight; achievement of HbA1c < 7% target; safety events
			non-Asian: 80.7%	vildagliptin 50 mg bid	83	24 weeks	2.4 (3.2)	50.2 (12.7)	43.4	8.4 (0.9)	32.2 (6.0)	
		Locations: US, India, Slovakia	non-Asian: 82.4%	vildagliptin 100 mg qd	91	24 weeks	2.1 (2.9)	52.0 (11.7)	46.2	8.3 (0.8)	31.9 (5.0)	
			non-Asian: 82.6%	placebo	92	24 weeks	2.5 (3.7)	52.0 (12.0)	45.7	8.5 (0.8)	32.7 (6.4)	
59	Scherbaum, et al 2008	Design: single-blind, randomized, placebo-controlled study for drug-naive T2D patients	non-Asian: 100%	vildagliptin 50 mg qd	68	108 weeks	2.1 (2.1)	63.1 (9.6)	39.7	6.6 (0.4)	30.4 (4.7)	changes from baseline in HbA1c, FPG, body weight; safety events
		Locations: Finland, France, Germany,	non-Asian:	placebo	63	108 weeks	2.5 (2.6)	63.2 (10.0)	41.3	6.7 (0.4)	30.1 (4.5)	

		Romania, Spain, Sweden	100%									
60	Foley, et al 2011	Design: double-blind, randomized, placebo-controlled study for drug-naive T2D patients	non-Asian: 96.6%	vildagliptin 100 mg qd	29	52 weeks	1.4 (2.8)	57.4 (9.4)	41.4	6.0 (0.7)	29.9 (4.9)	changes from baseline in HbA1c, FPG; safety events
		Location: Netherlands	non-Asian: 90.0%	placebo	30	52 weeks	0.6 (1.1)	57.0 (6.7)	40.0	6.0 (0.7)	29.2 (4.4)	
61	Haak, et al 2012	Design: randomized, double-blind, placebo-controlled study for drug-naive T2D patients	non-Asian: 68.3%	linagliptin 5 mg qd	142	24 weeks	25.7% patients with diabetes > 5 years	56.2 (10.8)	43.7	8.7 (1.0)	29.0 (4.7)	changes from baseline in HbA1c, FPG; achievements of HbA1c < 7% and < 6.5% targets; safety events
			non-Asian: 64.6%	metformin 0.5 g bid	144	24 weeks		52.9 (10.4)	43.1	8.7 (0.9)	28.9 (4.8)	
			non-Asian: 64.0%	metformin 1 g bid	147	24 weeks		55.2 (10.6)	46.9	8.5 (0.9)	29.5 (5.3)	
			non-Asian: 63.9%	placebo	72	24 weeks		55.7 (11.0)	50.0	8.7 (1.0)	28.6 (5.2)	
		Locations: 14 countries	non-Asian: 74.1%	linagliptin 2.5 mg qd + metformin 0.5 g bid	143	24 weeks		55.6 (11.2)	49.0	8.7 (1.0)	29.7 (5.3)	
			non-Asian: 66.4%	linagliptin 2.5 mg qd + metformin 1 g bid	143	24 weeks		56.4 (10.7)	46.2	8.7 (1.0)	28.6 (4.8)	
**DPP-4 inhibitors versus sulfonylureas (n = 1)**												
62	Hartley, et al 2015	Design: randomized, double-blind, active-controlled study for elderly T2D patients with diet/exercise therapy	non-Asian: 97.5%	sitagliptin 50—100 mg qd	197	30 weeks	8.0 (5.6)	70.6 (4.8)	52.8	7.8 (0.7)	29.7 (4.0)	changes from baseline in HbA1c, FPG, body weight; achievements of HbA1c < 7% and < 6.5% targets; safety events
		Locations: 13 countries	non-Asian: 93.7%	glimepiride 1—6 mg qd	191	30 weeks	9.4 (7.3)	70.8 (4.9)	59.7	7.8 (0.7)	29.7 (5.1)	
**DPP-4 inhibitors versus metformin or/and DPP-4 inhibitors as add-on drugs (n = 7)**												
63	Mu, et al 2017	Design: randomized, double-blind, active-controlled study for drug-naive T2D patients	Asian: 100%	linagliptin 5 mg qd	147	24 weeks	81% patients with diabetes < 1 year	50.8 (10.5)	48.3	8.7 (0.9)	26.2 (3.9)	changes from baseline in HbA1c, FPG, PPG, body weight; achievements of HbA1c < 7% and < 6.5% targets; safety events
			Asian: 100%	metformin 0.5 g bid	145	24 weeks		52.1 (9.6)	37.2	8.7 (1.1)	25.8 (3.3)	
			Asian: 100%	metformin 1 g bid	144	24 weeks		51.4 (10.4)	36.8	8.6 (1.0)	26.1 (3.3)	
		Locations: China, Malaysia, Philippines, Vietnam	Asian: 100%	linagliptin 2.5 mg qd + metformin 0.5 g bid	147	24 weeks		51.4 (10.2)	37.4	8.7 (0.9)	26.0 (3.6)	
			Asian: 100%	linagliptin 2.5 mg qd + metformin 1 g bid	147	24 weeks		50.7 (9.4)	40.8	8.7 (1.0)	26.0 (3.7)	
64	Williams-Herman, et al 2010	Design: randomized, double-blind, active-controlled study for	non-Asian: 92.6%	sitagliptin 100 mg qd	52	104 weeks	3.7 (4.9)	54.1 (9.1)	42.2	8.5 (0.9)	30.3 (5.5)	changes from baseline in HbA1c, FPG,

		T2D patients on diet and exercise	non-Asian: 92.3%	metformin 0.5 g bid	65	104 weeks	4.0 (3.9)	55.9 (8.9)	54	8.6 (0.9)	32.2 (6.9)	PPG, body weight, insulin level, lipids profile; achievement of HbA1c < 7% target; safety events
			non-Asian: 94.5%	metformin 1 g bid	88	104 weeks	3.9 (4.0)	54.3 (9.9)	56	8.5 (0.8)	31.9 (7.1)	
		Locations: 18 countries	non-Asian: 95.3%	sitagliptin 50 mg bid + metformin 0.5 g bid	100	104 weeks	3.7 (4.3)	54.5 (9.5)	50	8.7 (0.9)	31.6 (7.3)	
			non-Asian: 94.0%	sitagliptin 50 mg bid + metformin 1 g bid	107	104 weeks	4.4 (4.2)	53.9 (8.6)	63	8.6 (1.0)	31.4 (6.0)	
65	Russell-Jones, et al 2012	Design: multicentre, randomized, double-blind, active-controlled study for drug-naive T2D patients	non-Asian: 82.8%	sitagliptin 100 mg qd	163	26 weeks	2.7 (3.7)	52 (11)	42.3	8.5 (1.3)	31.8 (5.4)	changes from baseline in HbA1c, FPG, body weight; achievements of HbA1c < 7% and < 6.5% targets; safety events
			non-Asian: 83.4%	metformin 2 g/day	246	26 weeks	2.6 (3.6)	54 (11)	37.4	8.6 (1.2)	30.7 (5.5)	
		Locations: 21 countries	non-Asian: 81.6%	pioglitazone 45 mg/day	163	26 weeks	2.7 (3.7)	55 (11)	59.5	8.5 (1.2)	31.1 (5.3)	
66	Schwerzer, et al 2009	Design: randomized, double-blind, active-controlled study for drug-naïve T2D patients	non-Asian: 81.1%	vildagliptin 100 mg qd	169	24 weeks	2.9 (4.2)	71.6 (5.2)	55.6	7.8 (0.6)	29.8 (4.4)	changes from baseline in HbA1c, FPG, body weight; achievements of HbA1c < 7% and < 6.5% targets; safety events
		Locations: 14 countries	non-Asian: 78.3%	metformin 1.5 g/day	166	24 weeks	3.0 (4.7)	70.2 (5.1)	47.0	7.7 (0.6)	29.4 (4.6)	
67	Li, et al 2014	Design: randomized, active-controlled study for T2D patients	Asian: 100%	saxagliptin 5 mg qd	48	24 weeks	—	63.4 (5.1)	43.8	9.3 (1.6)	—	changes from baseline in HbA1c, FPG, PPG
		Location: China	Asian: 100%	metformin 0.5 g tid	48	24 weeks				9.7 (1.5)		
68	Wan, et al 2015	Design: randomized, active-controlled study for T2D patients	Asian: 100%	saxagliptin 5 mg qd	36	24 weeks	—	55.5 (16.38)	54.8	8.11 (1.53)	—	changes from baseline in HbA1c, FPG, lipids profile
			Asian: 100%	metformin 1 g/day	33	24 weeks				8.49 (1.67)		
		Location: China	Asian: 100%	saxagliptin 5 mg qd + metformin 1 g qd	35	24 weeks				8.24 (1.71)		
69	Li, et al 2014	Design: randomized, active-controlled study for newly-diagnosed T2D patients with metformin treatment	Asian: 100%	saxagliptin 5 mg qd + metformin 1 g/day	33	24 weeks	newly-diagnosed T2D patients	56.48 (9.13)	51.5	7.55 (0.74)	28.03 (2.61)	changes from baseline in HbA1c, FPG, PPG, BMI, insulin level; safety events
		Location: China	Asian: 100%	metformin 1 g/day	33	24 weeks		52.79 (11.72)	42.4	7.36 (0.74)	27.48 (2.94)	
**DPP-4 inhibitors versus thiazolidinediones (n = 1)**												
70	Nauck, et al 2016	Design: randomized, double-blind, active-controlled study for T2D patients	non-Asian: 97.0%	linagliptin 5 mg qd	135	30 weeks	70% paients with diabetes < 5 years	56.0 (10.4)	38.5	8.0 (0.9)	32.7 (5.3)	changes from baseline in HbA1c, FPG, PPG; achievements

			non-Asian: 97.7%	pioglitazone 15 mg/day	131	30 weeks		56.3 (10.4)	44.3	8.3 (0.9)	32.3 (5.6)	of HbA1c < 7% and < 6.5% targets; safety events
		Locations: Estonia, Germany, Latvia, Spain, U.K., U.S.	non-Asian: 97.9%	pioglitazone 30 mg/day	140	30 weeks		57.0 (11.5)	47.9	8.0 (0.9)	32.2 (5.3)	
			non-Asian: 98.6%	pioglitazone 45 mg/day	138	30 weeks		56.5 (11.0)	47.8	8.1 (0.9)	33.9 (5.5)	
**DPP-4 inhibitors versus DPP-4 inhibitors (n = 5)**												
71	Jin, et al 2015	Design: multicentre, randomized, double-blind, active-controlled study for T2D patients with metformin treatment	Asian: 100%	sitagliptin 100 mg qd + metformin	86	24 weeks	7.45 (5.33)	56.42 (9.83)	51.2	7.56 (0.63)	25.67 (3.66)	changes from baseline in HbA1c, FPG, body weight, insulin level, lipids profile; achievements of HbA1c < 7% and < 6.5% targets; safety events
		Location: Korea	Asian: 100%	anagliptin 100 mg bid + metformin	89	24 weeks	8.49 (5.88)	56.78 (8.50)	48.3	7.71 (0.71)	24.93 (3.16)	
72	Hong, et al 2017	Design: multicentre, randomized, double-blind, active-controlled study for T2D patients with metformin treatment	Asian: 100%	sitagliptin 100 mg qd + metformin	110	24 weeks	7.9 (4.9)	57.3 (9.3)	52.7	7.44 (0.73)	25.3 (2.7)	changes from baseline in HbA1c, FPG, body weight, insulin level, lipids profile; achievement of HbA1c < 6.5% target; safety events
		Location: Korea	Asian: 100%	evogliptin 5 mg qd + metformin	112	24 weeks	8.5 (5.5)	57.6 (9.4)	54.5	7.44 (0.73)	25.6 (3.7)	
73	Rhee, et al 2013	Design: multicentre, randomized, double-blind, active-controlled study for T2D patients with metformin treatment	Asian: 100%	sitagliptin 100 mg qd + metformin	133	24 weeks	6.4 (4.86)	52.94 (10.5)	46.62	8.05 (0.82)	26.27 (3.64)	changes from baseline in HbA1c, FPG, PPG, insulin level, lipids profile; achievements of HbA1c < 7% and < 6.5% targets; safety events
			Asian: 100%	gemigliptin 25 mg bid _ metformin	136	24 weeks	6.33 (4.69)	51.88 (10.62)	50.00	8.07 (0.86)	25.91 (3.51)	
		Locations: Korea, India	Asian: 100%	gemigliptin 50 mg qd _ metformin	135	24 weeks	6.14 (5.05)	53.99 (8.57)	40.00	7.93 (0.75)	25.64 (3.35)	
74	Goldenberg, et al 2017	Design: multicentre, randomized, double-blind, active-controlled study for T2D patients with metformin treatment	non-Asian: 86.2%	sitagliptin 100 mg qd + metformin	320	24 weeks	7.5 (5.6)	58 (10)	45.3	7.5 (0.7)	31.3 (5.1)	changes from baseline in HbA1c, FPG; achievements of HbA1c < 7% and < 6.5% targets; safety events
		Locations: 13 countries	non-Asian: 90.1%	omarigliptin 25 mg qw + metformin	322	24 weeks	7.0 (4.5)	57 (10)	53.1	7.5 (0.8)	32.7 (6.1)	
75	Chen, et al 2016	Design: randomized, open-label, active-controlled study for T2D patients with metformin treatment	Asian: 100%	vildagliptin 50 mg bid + metformin + gliclazide	37	24 weeks	6.62 (2.38)	63.68 (6.33)	43.24	8.38 (0.69)	22.90 (3.09)	changes from baseline in HbA1c, FPG, PPG, BMI; safety events
		Location: China	Asian: 100%	saxagliptin 5 mg qd + metformin + gliclazide	36	24 weeks	7.33 (2.26)	62.11 (6.75)	47.22	8.33 (0.67)	24.14 (4.27)	

SD, standard deviation; HbA1c, hemoglobin A1c; BMI, body mass index; DPP-4, dipeptidyl peptidase-4; T2D, type 2 diabetes; TID, thrice a day; BID, twice a day; FPG, fasting plasms glucose; QD, once a day; PPG, postprandial plasma glucose; NA, not applicable; QW, once a week; IQR, interquartile range; AM, ante meridiem; PM, post meridiem.
