# Supplementary material for: Acarbose With Comparable Glucose-Lowering but Superior Weight-Loss Efficacy to Dipeptidyl Peptidase-4 Inhibitors: A Systematic Review and Network Meta-Analysis of Randomized Controlled Trials
Source: Front Endocrinol (Lausanne). 2020 Jun 5;11:288. doi: 10.3389/fendo.2020.00288 (PMC7291873; doi:10.3389/fendo.2020.00288)
Supplement: Supplementary file 4 [file Table_3.PDF]

Table S3. Risk of bias assessment for the 75 included studies.

Study	Random sequence generation	Allocation concealment	Blinding of participants and personnel	Blinding outcome assessment	Incomplete outcome data	Selective reporting	Other bias
Pan, et al 2008	low risk	low risk	low risk	low risk	low risk	low risk	low risk
Wang, et al 2015	unclear	unclear	unclear	unclear	high risk	low risk	high risk
Du, et al 2017	low risk	unclear	high risk	high risk	low risk	low risk	unclear
Bao, et al 2014	high risk	unclear	high risk	high risk	high risk	low risk	unclear
Guo, et al 2015	unclear	unclear	high risk	high risk	high risk	low risk	high risk
Li, et al 2016	low risk	low risk	high risk	high risk	high risk	low risk	unclear
Xue, et al 2016	unclear	unclear	high risk	high risk	high risk	unclear	high risk
Zhang, et al 2016	low risk	low risk	high risk	high risk	low risk	unclear	low risk
Gao, et al 2015	low risk	low risk	high risk	high risk	low risk	low risk	low risk
Su, et al 2016	low risk	unclear	unclear	high risk	low risk	low risk	unclear
Li, et al 2016	unclear	unclear	unclear	unclear	high risk	unclear	high risk
Zhang, et al 2016	low risk	unclear	high risk	high risk	low risk	unclear	unclear
Duan, et al 2016	unclear	high risk	high risk	high risk	low risk	low risk	low risk
Hanefeld, et al 1991	low risk	low risk	low risk	low risk	high risk	unclear	unclear
Coniff, et al 1995	low risk	low risk	low risk	low risk	high risk	unclear	unclear
Hoffmann, et al 1994	low risk	low risk	low risk	low risk	high risk	unclear	unclear
Hoffmann, et al 1997	low risk	low risk	high risk	high risk	high risk	unclear	unclear
Chan, et al 1998	low risk	low risk	low risk	low risk	low risk	low risk	unclear
Holman, et al 1999	low risk	low risk	low risk	low risk	high risk	high risk	high risk
Josse, et al 2003	low risk	low risk	low risk	low risk	low risk	low risk	unclear
Kirkman, et al 2006	low risk	low risk	low risk	low risk	high risk	low risk	unclear
Wolever, et al 1997	low risk	low risk	low risk	low risk	low risk	low risk	high risk
Zheng, et al 1995	low risk	low risk	low risk	low risk	high risk	low risk	low risk
Fischer, et al 1998	low risk	low risk	low risk	low risk	high risk	low risk	low risk
Wu, et al 2003	low risk	low risk	low risk	low risk	high risk	low risk	low risk
Hasche, et al 1999	low risk	low risk	low risk	low risk	low risk	low risk	low risk
Braun, et al 1996	low risk	low risk	low risk	low risk	high risk	low risk	low risk
Kovacevic, et al 1997	low risk	low risk	low risk	low risk	high risk	low risk	high risk
Hu, et al 2012	low risk	unclear	unclear	unclear	low risk	low risk	low risk
Yang, et al 2013	low risk	high risk	high risk	high risk	low risk	low risk	low risk
Wang, et al 2011	low risk	unclear	unclear	unclear	low risk	low risk	low risk
Rong, et al 2008	low risk	unclear	unclear	unclear	high risk	low risk	high risk
Zhu, et al 2011	low risk	unclear	unclear	unclear	high risk	low risk	high risk
G__ke, et al 2002	low risk	high risk	high risk	high risk	low risk	low risk	low risk
E, et al 2015	low risk	high risk	high risk	high risk	low risk	low risk	low risk
Defronzo, et al 2008	low risk	low risk	low risk	low risk	high risk	low risk	high risk
Inagaki, et al 2015	low risk	low risk	low risk	low risk	low risk	low risk	low risk
Ji, et al 2017	low risk	low risk	low risk	low risk	high risk	low risk	high risk
Yang, et al 2015	low risk	low risk	low risk	low risk	low risk	low risk	low risk
Park, et al 2017	low risk	low risk	low risk	low risk	high risk	low risk	low risk

Yang, et al 2012	low risk	low risk	low risk	low risk	high risk	low risk	high risk
Del Prato, et al 2011	low risk	low risk	low risk	low risk	low risk	low risk	high risk
Chen, et al 2015	low risk	low risk	low risk	low risk	high risk	low risk	high risk
Wu, et al 2015	low risk	low risk	low risk	low risk	low risk	low risk	low risk
Rosenstock, et al 2009	low risk	low risk	low risk	low risk	high risk	low risk	low risk
Frederich, et al 2012	low risk	low risk	high risk	high risk	high risk	low risk	high risk
Pan, et al 2012	low risk	low risk	low risk	low risk	low risk	low risk	low risk
Kumar, et al 2014	low risk	low risk	low risk	low risk	low risk	low risk	low risk
Aschner, et al 2006	low risk	low risk	low risk	low risk	high risk	low risk	low risk
Goldstein, et al 2007	low risk	low risk	low risk	low risk	high risk	low risk	low risk
Barzilai, et al 2011	low risk	low risk	low risk	low risk	high risk	low risk	low risk
Ji, et al 2016	low risk	low risk	low risk	low risk	high risk	low risk	low risk
Gantz, et al 2017	low risk	low risk	low risk	low risk	low risk	low risk	low risk
Gupta, et al 2017	low risk	low risk	low risk	low risk	high risk	low risk	low risk
Zhao, et al 2017	low risk	low risk	low risk	low risk	low risk	low risk	low risk
Hong, et al 2016	low risk	low risk	low risk	low risk	high risk	low risk	low risk
Dejager, et al 2007	low risk	low risk	low risk	low risk	high risk	low risk	low risk
Pi-Sunyer, et al 2007	low risk	low risk	low risk	low risk	high risk	low risk	low risk
Scherbaum, et al 2008	low risk	low risk	high risk	high risk	low risk	low risk	low risk
Foley, et al 2011	low risk	low risk	low risk	low risk	high risk	low risk	high risk
Haak, et al 2012	low risk	low risk	low risk	low risk	high risk	low risk	low risk
Hartley, et al 2015	low risk	low risk	low risk	low risk	low risk	low risk	low risk
Schwerzer, et al 2009	low risk	low risk	low risk	low risk	high risk	low risk	low risk
Li, et al 2014	low risk	unclear	unclear	unclear	high risk	low risk	high risk
Russell-Jones, et al 2012	low risk	low risk	low risk	low risk	high risk	low risk	low risk
Nauck, et al 2016	low risk	low risk	low risk	low risk	high risk	low risk	low risk
Jin, et al 2015	low risk	low risk	low risk	low risk	low risk	low risk	low risk
Hong, et al 2017	low risk	low risk	low risk	low risk	low risk	low risk	low risk
Rhee, et al 2013	low risk	low risk	low risk	low risk	high risk	low risk	low risk
Goldenberg, et al 2017	low risk	low risk	low risk	low risk	high risk	low risk	low risk
Chen, et al 2016	low risk	high risk	high risk	high risk	high risk	low risk	high risk
Mu, et al 2017	low risk	low risk	low risk	low risk	high risk	low risk	low risk
Williams-Herman, et al 2010	low risk	low risk	low risk	low risk	high risk	low risk	low risk
Wan, et al 2015	low risk	unclear	unclear	unclear	high risk	low risk	high risk
Li, et al 2014	low risk	unclear	unclear	unclear	low risk	low risk	low risk
