# Supplementary material for: Acarbose With Comparable Glucose-Lowering but Superior Weight-Loss Efficacy to Dipeptidyl Peptidase-4 Inhibitors: A Systematic Review and Network Meta-Analysis of Randomized Controlled Trials
Source: Front Endocrinol (Lausanne). 2020 Jun 5;11:288. doi: 10.3389/fendo.2020.00288 (PMC7291873; doi:10.3389/fendo.2020.00288)
Supplement: Supplementary file 5 [file Table_4.PDF]

Table S4. Univariate meta-regression analysis results of 2hPG change in pair-wise meta-analysis.

	**Estimate**	**95% CI**	**p value**
Diabetes duration	-0.541	(-1.269, 0.188)	0.146
Treatment duration	0.077	(0.050, 0.104)	0.000
Age	0.074	(-0.021, 0.170)	0.128
Acarbose dosages	0.005	(0.001, 0.009)	0.011
Baseline HbA1c level	-0.375	(-0.955, 0.204)	0.204
Study with statistical significance	0.397	(-0.182, 0.975)	0.179

2hPG, 2-hour postprandial glucose; CI, confidence interval.
